# Altered brain connectivity in sudden unexpected death in epilepsy (SUDEP) revealed using resting-state fMRI

**DOI:** 10.1016/j.nicl.2019.102060

**Published:** 2019-10-28

**Authors:** Allen LA, Harper RM, Guye M, Kumar R, Ogren JA, Vos SB, Ourselin S, Scott CA, Lhatoo SD, Lemieux L, Diehl B

**Affiliations:** a Department of Clinical and Experimental Epilepsy, UCL Queen Square Institute of Neurology, London, UK; b Epilepsy Society MRI Unit, Chalfont St Peter, Buckinghamshire, UK; c The Center for SUDEP Research, National Institute of Neurological Disorders and Stroke, Bethesda, MD, USA; d UCLA Brain Research Institute, Los Angeles, CA, USA; e Department of Neurobiology, David Geffen School of Medicine at UCLA, Los Angeles, CA, USA; f Aix Marseille University, CNRS, CRMBM UMR 7339, Marseille, France; g Department of Anesthesiology, David Geffen School of Medicine at UCLA, Los Angeles, CA, USA; h Department of Radiological Sciences, David Geffen School of Medicine at UCLA, Los Angeles, CA, USA; i Department of Bioengineering, David Geffen School of Medicine at UCLA, Los Angeles, CA, USA; j Wellcome / EPSRC Centre Interventional and Surgical Sciences, UCL, London, UK; k Translational Imaging Group, Centre for Medical Image Computing, UCL, London, UK; l School of Biomedical Engineering and Imaging Sciences, St Thomas’ Hospital, King's College London, London, UK; m Department of Neurology, University of Texas Health Sciences Center at Houston, Houston, TX, USA

**Keywords:** SUDEP, Rs-fmri, Graph theory, Amygdala, Thalamus

## Abstract

The circumstances surrounding SUDEP suggest autonomic or respiratory collapse, implying central failure of regulation or recovery. Characterisation of the communication among brain areas mediating such processes may shed light on mechanisms and noninvasively indicate risk.

We used rs-fMRI to examine network properties among brain structures in people with epilepsy who suffered SUDEP (*n* = 8) over an 8-year follow-up period, compared with matched high- and low-risk subjects (*n* = 16/group) who did not suffer SUDEP during that period, and a group of healthy controls (*n* = 16). Network analysis was employed to explore connectivity within a ‘regulatory-subnetwork’ of brain regions involved in autonomic and respiratory regulation, and over the whole-brain.

Modularity, the extent of network organization into separate modules, was significantly reduced in the regulatory-subnetwork, and the whole-brain, in SUDEP and high-risk. Increased participation, a local measure of inter-modular belonging, was evident in SUDEP and high-risk groups, particularly among thalamic structures. The medial prefrontal thalamus was increased in SUDEP compared with all other control groups, including high-risk. Patterns of hub topology were similar in SUDEP and high-risk, but were more extensive in low-risk patients, who displayed greater hub prevalence and a radical reorganization of hubs in the subnetwork.

SUDEP is associated with reduced functional organization among cortical and sub-cortical brain regions mediating autonomic and respiratory regulation. Living high-risk subjects demonstrated similar patterns, suggesting such network measures may provide prospective risk-indicating value, though a crucial difference between SUDEP and high-risk was altered connectivity of the medial thalamus in SUDEP, which was also elevated compared with all sub-groups. Disturbed thalamic connectivity may reflect a potential non-invasive marker of elevated SUDEP risk.

## Highlights

• The functional architecture among regulatory structures, and the whole brain, is less modular in confirmed cases of SUDEP and those at high-risk.
• Altered functional organisation may mean potential impairment of communication among key regulatory circuits.
• SUDEP is associated with regional connectivity disruptions among cortical and sub-cortical regulatory sites.
• Medial thalamic connectivity was significantly altered in SUDEP compared with all control groups, including those at high-risk.
• Increases in the number, and a shift in organisation, of hubs appears to relate to lower mortality risk.

## 1 Introduction

Non-invasive determination of risk for sudden unexpected death in epilepsy (SUDEP) and unearthing of processes leading to that outcome are major goals in the epilepsy field, since the scenario is the most common cause of premature death in people with epilepsy, with a 20-fold increase over that of sudden death in the general population (Surges and Sander, 2012). Although the precise mechanisms of SUDEP remain elusive, circumstances surrounding the fatal event suggest a sudden cardiovascular collapse or cessation of respiratory efforts (Ryvlin et al., 2013; Massey et al., 2014) implying a failure of central regulatory control.

Resting-state functional magnetic resonance imaging (rs-fMRI) studies show that patients at high risk of SUDEP show altered functional connectivity between key cortical and sub-cortical autonomic and respiratory control regions (Allen et al., 2017; Tang et al., 2014). Disrupted functional interactions among these regions along the cortico-diencephalic-brainstem pathway for cardiovascular and breathing control are suspected of contributing to SUDEP by interfering with normal control of blood pressure or breathing. The rs-fMRI methodology allows non-invasive assessment of alterations to underlying functional neural pathways.

Structural imaging studies of patients who later succumbed to SUDEP, and those at greatest risk (those who experience generalised tonic-clonic seizures; GTCS) reveal morphologic changes in key autonomic brain structures (Wandschneider, 2015; Ogren et al., 2018); determining accompanying functional network disturbances should provide new insights into failing mechanisms. Previous studies assessing functional interactions between areas mediating autonomic and respiratory functions focused on specific epilepsy subgroups, e.g., temporal lobe epilepsy, in risk-stratified living patients (Allen et al., 2017; Tang et al., 2014) and no confirmed or suspected SUDEP cases have been studied to date.

We characterized noninvasively the nature of functional interactions amongst a network of brain sites known to mediate cardiovascular and breathing control in confirmed SUDEP cases. We used rs-fMRI and network analysis procedures in patient groups that included SUDEP cases, patients at high and low SUDEP risk, and matched healthy controls. The goal was to provide insights into potential mechanisms of failure and suggest possible non-invasive means to evaluate risk for SUDEP.

## 2 Methods

### 2.1 Subjects

Cases of SUDEP, high- and low-risk patients, and healthy controls were selected from an ongoing investigation into the fMRI correlates of interictal epileptiform discharges (Coan et al., 2016) with a case ascertainment period between 2005 and 2014. During this time, scanner hardware and software remained unchanged. The inclusion criteria were the availability of: (1) a resting-state EEG-fMRI scan, and (2) a high-resolution T1-weighted scan. The exclusion criteria were: (1) large brain lesions or previous neurosurgery (we considered large to be anything greater than a small area of FCD or sclerosis – i.e. tumours, cavernomas etc.); 2) incomplete clinical or imaging data (e.g., abandoned scans); and (3) excessive head movement during the EEG-fMRI scan (inter-scan displacement exceeding 3 mm in any direction).

We searched the database for deaths, by querying each subject's medical record profile on a local clinical records database, and confirming these with death certificates. Of 12 deaths, nine were identified as SUDEP, one of which was excluded due to the presence of a large brain lesion (previous neurosurgery). The resulting eight SUDEP cases (4 males, mean age 26.6 ± 6.1; see Table 1 for patient characteristics) were then classified as either probable or definite SUDEP based on established criteria (Nashef et al., 2012).

**Table 1:** Clinical characteristics of the SUDEP cases. *M* = male, *F* = female, def = definite, prob = probable, JME = juvenile myoclonic epilepsy, *L* = left, *R* = right, FCD = focal cortical dysplasia, hem = hemisphere.

Case	SUDEP class	Scan - death interval (years)	Epilepsy syndrome	Disease duration (years)	GTCS per month	MRI findings
01 (**M**)	*Def*	*3*	*Focal, L frontal*	*18*	*0.75*	*Normal*
02 (**M**)	*Def*	*6*	*JME*	*2*	*10*	*Normal*
03 (**F**)	*Prob*	*2*	*JME*	*6*	*1.5*	*Normal*
04 (**M**)	*Def*	*6*	*Focal, L hem*	*29*	*1*	*L parietal ischaemia*
05 (**F**)	*Def*	*6*	*Focal, L frontal*	*30*	*2*	*L frontal FCD*
06 (**F**)	*Prob*	*8*	*Focal, R parietal*	*10*	*1*	*R parietal FCD*
07 (**M**)	*Prob*	*2*	*Focal, L temporal*	*20*	*2*	*L anterior temporal FCD*
08 (**F**)	*Prob*	*6*	*Focal, L hem*	*18*	*5*	*Normal*

We matched each SUDEP case as closely as possible with 2 high-risk and 2 low-risk patients, based on epilepsy syndrome and localization, disease duration, age, sex and lesion pathology. All clinical information used for risk stratification and subject-matching was obtained from multidisciplinary team meeting reports and clinic letters at the time closest to the rs-fMRI scan. We chose to classify the matched patients according to a single criterion based on close examination of the SUDEP cases in our cohort: Experiencing more than 3 GTCS per year, which has been found to be the most predictive SUDEP risk factor (DeGiorgio et al., 2017; et al. 2017; Hesdorffer et al., 2012) and was the only common clinical factor in the SUDEP cases in our cohort. Thus, high-risk patients were defined as those experiencing more than 3 GTCS per year. Since SUDEP is dominantly a GTCS-related event (Lamberts et al., 2012) low-risk patients were those not experiencing any GTCS. In addition to living patient controls, each SUDEP case was matched to two healthy controls, of comparable age and same sex (individual patient and healthy control characteristics are shown in Supplementary Table S1).

The maximum time between scan and death for SUDEP cases was eight years. As such, we defined an eight-year follow up period for high-risk and low-risk subjects, to confirm survivorship. This was carried out by referring to patient status and clinical letters up to eight years following the scan. Four sub-groups resulted from the above procedures; SUDEP within eight years (*n* = 8), high-risk/no SUDEP within eight years (*n* = 16), low-risk/no SUDEP within eight years (*n* = 16) and healthy controls (*n* = 16). These groups were taken forward for further analysis of rs-fMRI data and inter-group comparisons (group demographics and clinical details are shown in supplementary Table S2). The study was approved by the National Research Ethics Committee (United Kingdom; 04/Q0512/77 and 14/SW/0021) and all patients gave written informed consent.

## 3 Magnetic resonance imaging acquisition

### 3.1 Resting-state fMRI

Scanning was performed at the Epilepsy Society (Chalfont St Peter, Buckinghamshire, UK) on a 3.0 Tesla GE (Signa Excite HDX) scanner. A 10-minute (200 vol) resting-state EEG-fMRI scan was collected for each subject with the following characteristics: repetition time (TR) = 3000 ms, echo time (TE) = 30 ms; flip angle = 90º, matrix size = 64 × 64, field of view (FOV) = 24 × 24 cm, slice thickness = 3 mm, 44 slices, voxel size = 3 mm^3^). Subjects were instructed to keep their eyes closed, avoid falling asleep, and not think about anything in particular. A 64-channel EEG was recorded during the fMRI scanning using an MRI-compatible amplifier and cap (Brain Products GmbH, Gilching, Germany). In this study, the EEG recordings were used solely to record occurrence of interictal epileptiform discharges.

### 3.2 Structural MRI

A single high-resolution T1-weighted image was also acquired immediately before rs-fMRI collection using a FSPGR (fast spoiled gradient recalled echo) sequence, with the following parameters: TR/TE = 8.10/3.2, 24 cm FOV, 100 slices, slice thickness = 1.5 mm, with a matrix size of 256 × 160 for a voxel size of 1 × 1 × 1.5 mm.

## 4 Data processing

### 4.1 Rs-fMRI pre-processing

Following routine rs-fMRI pre-processing steps (see Methods Section 2i in Supplementary Material for detailed descriptions), time-courses from 246 brain regions were extracted for each subject using the Brainnetome atlas (BNA; Fan et al., 2016). The BNA is composed of 7 ‘lobes’ (frontal, temporal, parietal, insular, cingulate, occipital and sub-cortical nuclei), with a total of 24 ‘sub-lobes’ (or gyri), sub-divided into a total of 246 regions, and was selected because of its high level of regional subdivision and its anatomical, structural and functional relevance. The maximal overlap discrete wavelet transform (MODWT) was used to obtain coefficients from scale 2 of the wavelet decomposition which, in our data, corresponded to the frequency range 0.03~0.06 Hz, since gray matter-derived network properties are most are most salient within this frequency range (Achard et al., 2006).

### 4.2 Regulatory subnetwork: ROI selection

The regulatory subnetwork was constructed from regions which play a significant role in cardiovascular and respiratory control, on the assumption that the fatal SUDEP event develops from a blood pressure collapse, significant arrhythmia, or failed respiratory efforts resulting in hypoxia (Massey et al., 2014). Regions of interest (ROIs) were selected from the BNA in a systematic fashion by including all regions within the structures belonging to lobes known to be associated with autonomic and respiratory functions, namely the cortico-diencephalic-brainstem pathways for autonomic and respiratory control (Macey et al., 2016; Shoemaker and Goswami, 2015; Loewy, 1991). This resulted in seventy-four ROIs: medial/orbito-frontal cortex (12 regions), insular cortex (12) and cingulate cortex (14), and 8 medial temporal structures (4 amygdala, 4 hippocampus), 12 basal ganglia and 16 thalamic regions (see Table S3 for details).

### 4.3 Network construction and analysis

The wavelet coefficients obtained from the rs-fMRI pre-processing were used to construct a whole-brain (246 ROI, or network “nodes”) and a regulatory network (74 nodes) for each subject. The resulting weighted networks (or “graphs”) were thresholded, using a minimal spanning tree approach, at network sparsities (proportion of connections) ranging from 50% to 5% in decrements of 1% and binarized (Alexander-Bloch et al., 2010). This process yielded a series of 46 binary undirected (involving non-directional connections) networks per subject, on which network measures were computed (network construction and graph theoretical measures are outlined in Methods Section (2ii) of the Supplementary Material). We computed four graph analytical measures, described earlier (Rubinov and Sporns, 2010) to explore various network properties, under the rationale that SUDEP may be related to altered connectivity and networking among regulatory structures, and across the entire brain. Network measures are conceptually described below; for more detailed, and mathematical, descriptions of these measures, see supplementary material (2. supplementary methods).

#### 4.3.1 Network modularity

Under our hypothesis that SUDEP may be linked to altered organization, and therefore communication, among brain regions involved in regulatory processes as well as the whole brain, we investigated modularity. Modularity is a network-wide assessment of how well a network can be sub-divided into clearly delineated groups (or modules) – a measure of how well-organised networks are.

#### 4.3.2 Nodal participation

Secondly, we computed participation, to understand if any particular brain regions exhibiting altered inter-modular communication are linked to SUDEP and/or elevated risk. The participation coefficient is a nodal measure associated with modularity, and assesses the extent to which a given region is connected to the other modules in the network. A node with high participation will have an equivalent number of connections to all the modules in the network. For a node with low participation, however, a larger proportion of its connections will lie within its own module. Participation is a measure of the diversity of a node, i.e., how much it ‘participates’ in other modules.

#### 4.3.3 Nodal degree centrality (DC)

We explored degree centrality (DC) to investigate whether differences in connectivity of specific regions, with the rest of the sub-network and whole-brain, may be related to SUDEP. DC is a nodal network measure, which can be used to explore ‘hubness’ – the tendency of real-world complex systems, such as human brain networks, to be organised around highly-connected hubs (Achard et al., 2006). DC is simply defined as the number of connections incident upon a node (after thresholding connection strength). The greater the number of connections belonging to a node, the greater its connectivity.

#### 4.3.4 Hub prevalence and hub distribution index (HDI)

Hubs are highly connected brain areas, i.e. regions having a greater number of connections (as can be assessed with DC), and are a key feature of human structural and functional brain architecture (Hagmann et al., 2008). We carried out hub analysis to determine whether the number (prevalence) or organisation (distribution) of hubs differed across groups, and whether these features may hold risk-indicating value. This was explored for both the whole-brain and subnetwork.

To assess changes in the number of hubs (hub prevalence) across groups, we first calculated the number of hubs belonging to each subject. This was performed by averaging DC across all network sparsities for each region, and defining hub nodes as regions for which DC was one standard deviation above the mean (of all regions) for a given subject (Agosta et al., 2013). The number of hubs was then compared across groups, as per statistics below.

To determine potential changes in hub organisation across groups, we calculated the hub distribution index (HDI). The HDI (Achard et al., 2012) characterizes the distribution of hubs within an individual (i.e. single patient) network, relative to a normative (healthy) group network. Specifically, the HDI is a relative measure of regional topology that quantifies the reorganization of hubs between two such networks. To estimate the HDI in a single subject, the healthy group mean degree (across subjects and network sparsities) for each region is simply subtracted from the mean degree (across sparsities) of the same region in an individual subject. The regional differences thus obtained are then plotted against the means of the healthy control group and a least square regression line is fitted to the data, per subject. Typically, when calculated for an individual healthy control, the data points on the plot scatter to form a positive horizontal slope, indicating that the degree of a given region in a given healthy control is close to the average degree of the same region for the remainder of the healthy group (Achard et al., 2012). A negative slope for an individual subject, however, indicates that high-degree regions (hubs) in the healthy group have become low-degree regions (non-hubs) in the test subject, demonstrating a reorganization of hubs (Achard et al., 2012; See supplementary Figure S1 for single subject example of HDI calculation). The gradient of the slope was measured for each subject and compared across patient groups, as described below in statistical analysis.

Due to inadequate whole-brain parcellation (incomplete T1 scan) in one subject, the whole-brain network analysis was carried out in 55 subjects (8 SUDEP, 15 high-risk, 16 low-risk and 16 healthy).

## 5 Statistical analysis of network measures

The following statistical analyses were carried out identically for the regulatory sub-network and whole-brain network.

### 5.1 Area under the curve (AUC) and permutation tests

The following calculations were carried out in Matlab: to establish between-group differences between each of the patient subgroups and the healthy controls for each network measure, we compared the area under the curve (AUC) across groups, using analysis of variance (ANOVA) and post-hoc two-sample permutation tests. The AUC was obtained by integrating the network measure values across all 46 sparsities, yielding one value/network measure, and per node for DC and participation, as opposed to one per sparsity, greatly reducing the number of statistical tests required for group comparisons and increasing power to detect differences across multiple sparsities (Zhang et al., 2017; Xu et al., 2016).

### 5.2 Regression of mean connectivity

Mean connectivity (often expressed as the mean Pearson *R* correlation, or mean strength, of a network) is known to influence regional functional connectivity and graph theoretical measurements as a result of individual variation, and explains variance related to physiology, sex, ageing and the degree of intra-scan movement (Geerligs et al., 2017). To account for this, we computed mean connectivity and regressed this from the AUC of resulting graph measures prior to statistical analysis, for both subnetwork and whole-brain analysis pipelines. Mean connectivity was calculated as the mean sum of weights of all regions, and was removed using linear regression (Geerligs et al., 2017).

### 5.3 Statistics: global measures

ANOVA tests were first performed to determine overall effects of group on each global network measure (modularity, hub prevalence and HDI). Post-hoc two-sample t-tests were then employed to explore significant differences between each of the groups.

### 5.4 Statistics: nodal measures

To explore regional differences among nodal graph measures (participation and degree centrality), the AUC of each measure per region was initially compared across groups using ANOVA. Upon observation of a significant main effect for a given region, post-hoc, two-sample permutation tests were carried out for every possible contrast combination. For each *t*-test contrast, members from each sample were randomly permuted upon each of 10,000 iterations. This step generates an empirical null distribution, from which p-values were obtained. After each contrast iteration, resulting p-values were corrected for multiple comparisons using the false discovery rate (FDR; Benjamini and Hochberg, 1995; Genovese et al., 2002); a correction which was unnecessary for the global measures (network modularity and HDI), since only one value per subject is calculated.

### 5.5 Correlation analysis

Correlations were carried out in IBM SPSS 25 to assess whether GTCS frequency correlated with the AUC of any of the graph measures computed on the subnetwork (modularity, participation, DC), as well as with hub distribution. SUDEP cases and high-risk subjects (*n* = 24) were considered as one group for this correlation analysis. *P*-values were FDR corrected as per group comparisons.

### 5.6 Accounting for epileptiform discharges during rs-fMRI

For each epilepsy subject (SUDEP, high-risk and low-risk groups), data were checked for interictal epileptiform discharges (IED; or ‘epileptic spikes’), by an experienced neurophysiologist. IEDs were visually counted and average (mean) IED counts (for each scan, per subject) were calculated and compared across patient groups using non-parametric statistical tests. Spearman's rank correlation coefficients were also carried out between IED counts and each of the network measures computed.

## 6 Results

### 6.1 Demographics and clinical data

Age and sex distributions for all groups at scan time were similar (Table S2). Epilepsy groups (SUDEP, high- and low-risk) were comparable for clinical variables, including localization and duration of epilepsy (Table S2.) All epilepsy groups had similar proportions of patients with a lesion identified on MRI (SUDEP: 50%, high-risk: 37.5%, low-risk: 50%).

### 6.2 Resting-state fMRI

The mean number of IEDs did not differ significantly between any of the patient groups, nor did it correlate significantly with the AUC of any of the network measures or hub distribution indices.

**Fig. 1:**
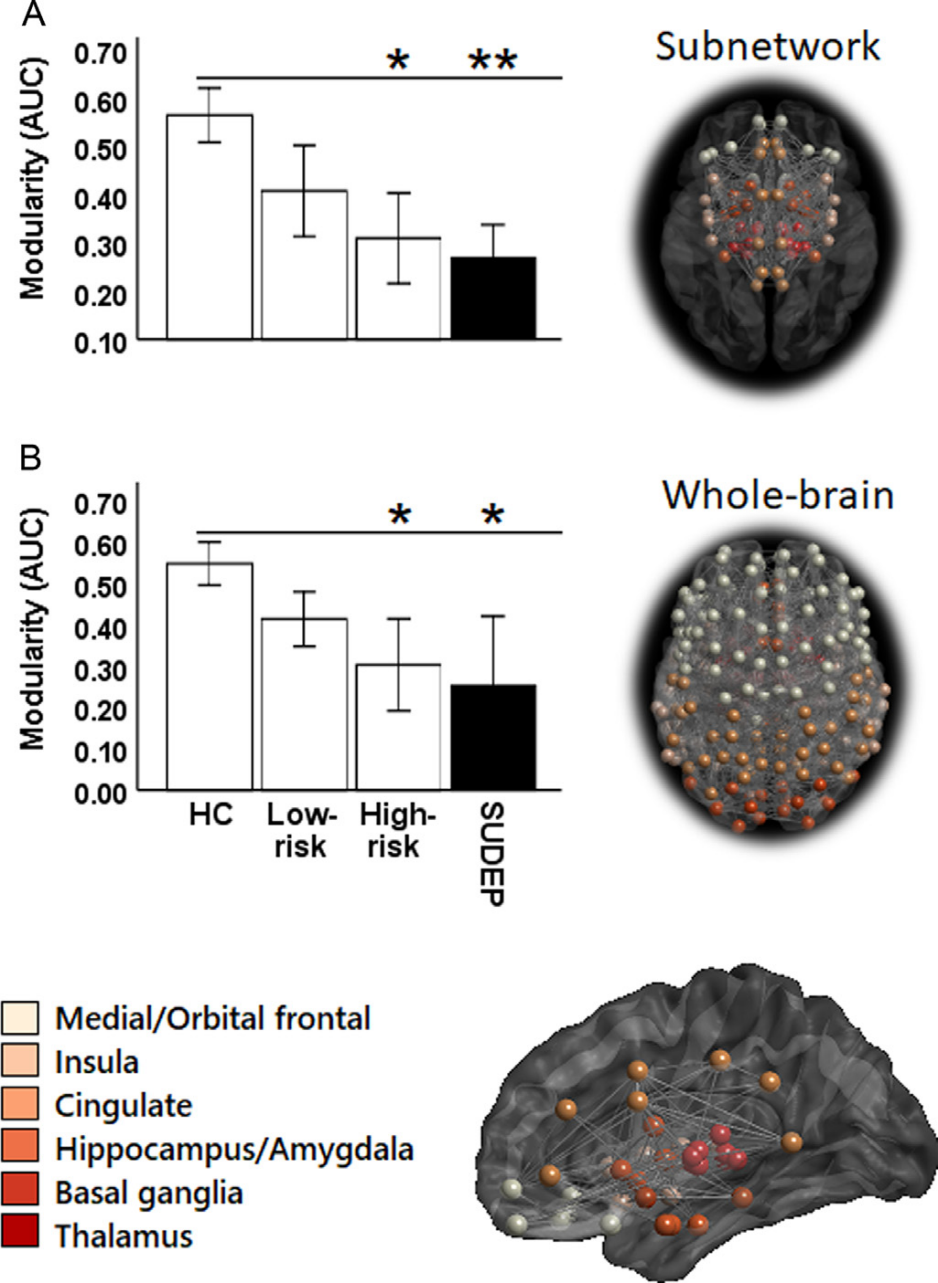
A: Reduced subnetwork modularity in low- and high-risk patients and cases of SUDEP, compared to healthy controls (HC). * (*p* < 0.05), ** (*p* = 0.005). Error bars = standard error (SE) +/−1. B: Reduced whole brain modularity in high-risk subjects and SUDEP cases compared with healthy controls (HC). Error bars = standard error (SE) +/−1. **Left:** bar graphs and **Right:** axial view of model network, with different colours representing module class. (For interpretation of the references to colour in this figure legend, the reader is referred to the web version of this article.)

A significant main effect of group was observed for modularity, as assessed with ANOVA (*F* (3,55) = 4.5, *p *= 0.004). Modularity of the regulatory subnetwork was significantly reduced in cases of SUDEP (*t *= 3.2, *p *= 0.004 [Cohen's *D *= 1.3]) and high-risk subjects (*t *= 2.4, *p *= 0.02 [*D *= 0.7]), compared with healthy controls (Fig. 1A). Subnetwork modularity was reduced in SUDEP when compared with low-risk subjects (*t *= 2.1, *p *= 0.03 [*D *= 0.6]). Similarly, whole-brain modularity was reduced in SUDEP cases (*t *= 2.2, *p *= 0.03 [*D *= 0.9]) and patients at high-risk (*t *= 2.1, *p *= 0.04 [*D *= 0.7]), compared with healthy controls (Fig. 1B).

**Fig. 2:**
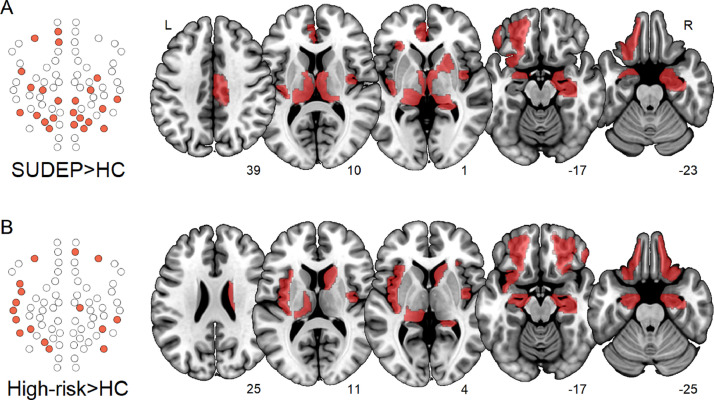
Increased participation (*p* < 0.05, FDR) in SUDEP (A) and high-risk (B) compared with healthy controls. **Left:** Network schematics of affected regions among the subnetwork in SUDEP and high-risk. **Right:** regions overlaid in red on a standard brain, with slice numbers below. (For interpretation of the references to colour in this figure legend, the reader is referred to the web version of this article.)

**Fig. 3:**
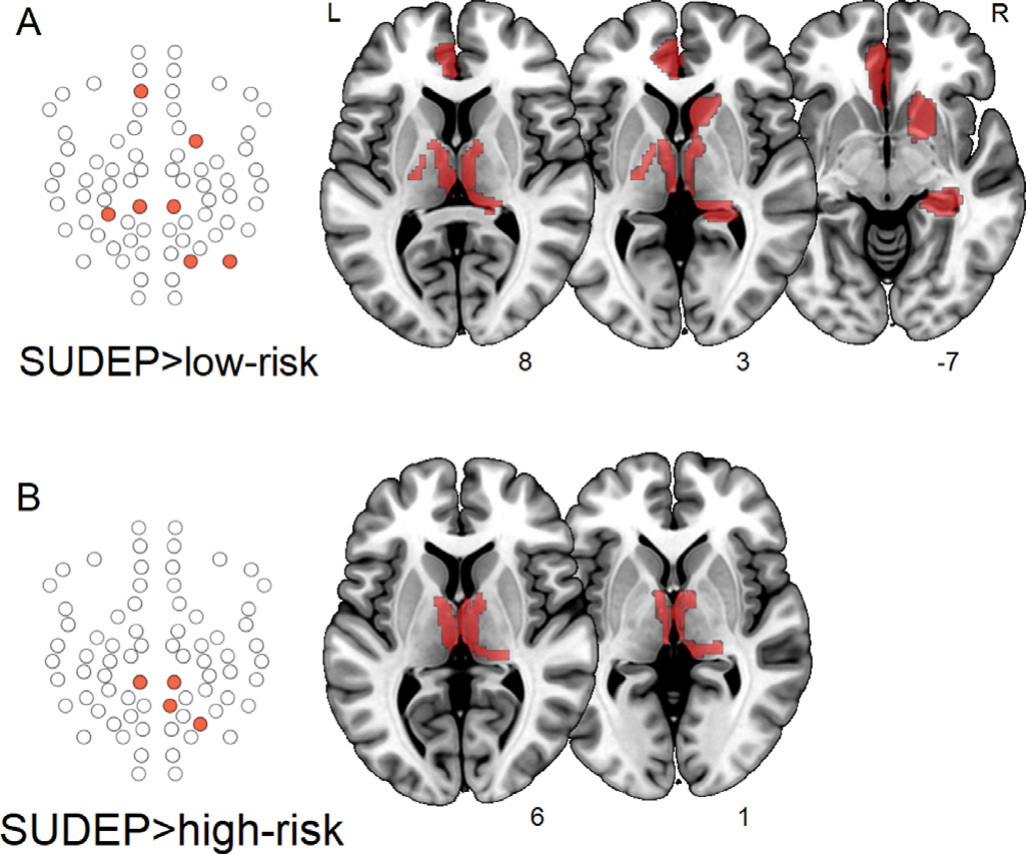
Increased participation (*p* < 0.05, uncorrected) in SUDEP compared with low-risk (A) and high-risk (B). **Left:** Network schematics of affected regions among the subnetwork in SUDEP and high-risk. **Right:** regions overlaid in red on a standard brain, with slice numbers below. (For interpretation of the references to colour in this figure legend, the reader is referred to the web version of this article.)

Compared with healthy controls, nodal participation within the subnetwork (the extent to which a region participated in modules outside its own) was significantly elevated across 16 regions in high-risk patients and 23 in SUDEP cases (Fig. 2). When compared with low-risk and high-risk subjects (at the uncorrected significance level), SUDEP cases additionally showed increased participation across 7 and 4 regions, respectively (Fig. 3). Participation of the medial prefrontal thalamus was significantly elevated compared with all other sub-groups, effect sizes of which were the largest of all nodal results. Details of all nodal results, including anatomical labels, and FDR-corrected p-values, can be found in Table S4.

**Fig. 4:**
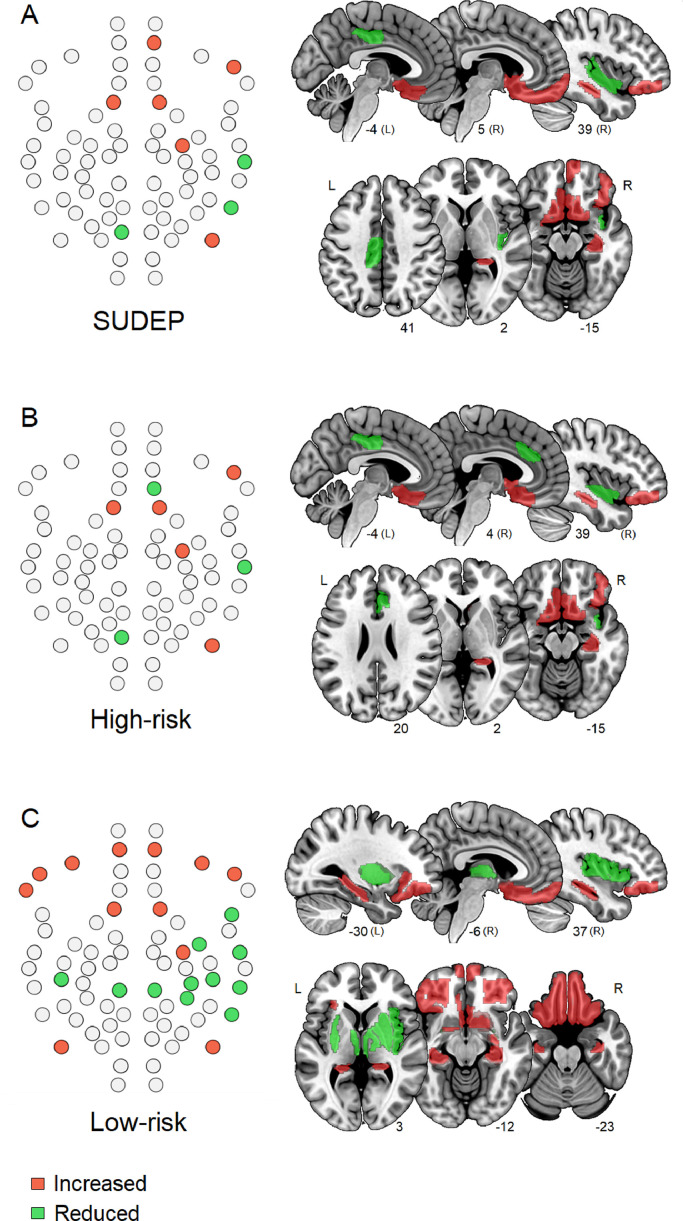
Increased (red) and reduced (green) degree centrality in SUDEP (A) high-risk (B) and low-risk (C) compared with healthy controls (*p* < 0.05, FDR). **Left:** regulatory subnetwork schematic, **right**: ROIs overlaid on standard brain. (For interpretation of the references to colour in this figure legend, the reader is referred to the web version of this article.)

Significant nodal elevations and reductions in DC (degree centrality, a measure of hubness) were observed in every patient group relative to healthy controls, for the subnetwork only. SUDEP and high-risk cases displayed similar patterns of change in DC, with reductions in the insula and cingulate, and increases in frontal and hippocampal structures (Fig. 4A, 3B). Low-risk subjects also showed increased and decreased DC vs controls, although these changes spanned a greater part of the network compared to high-risk and SUDEP cases (Fig. 4C; see Supplementary Table S5 and S6 for detailed affected nodes and FDR corrected p-values).

Effect sizes for regional participation and degree centrality group comparison results can be found in table S7 and S8, respectively.

Hub prevalence (the number of hubs calculated for each subject) among the subnetwork was significantly greater in low-risk subjects compared with SUDEP cases (*t *= 2.8, *p *= 0.01), and was highest in low-risk subjects overall (Supplementary Figure S2). For group means and effect sizes see Supplementary Table S9.

**Fig. 5:**
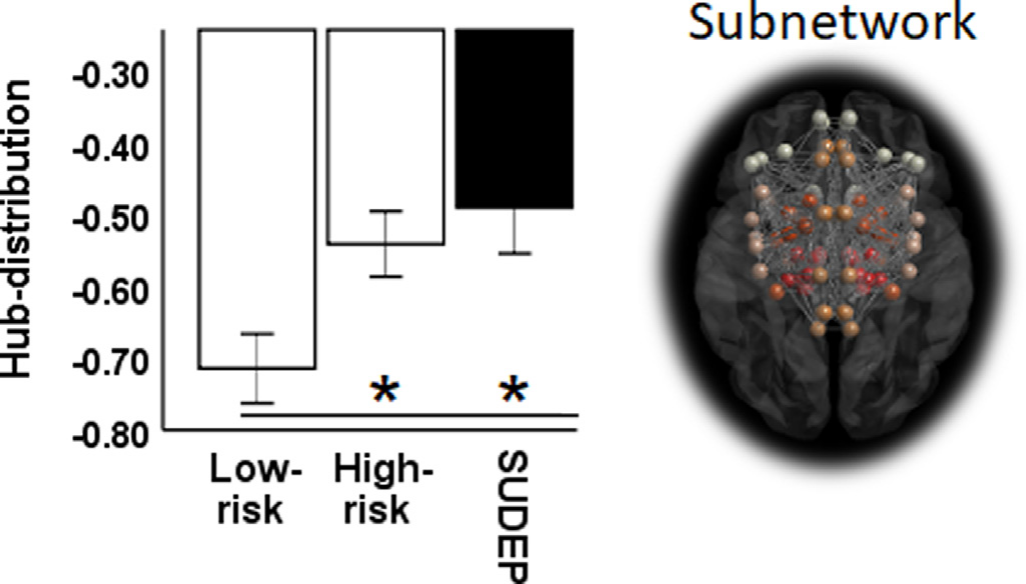
Greater reorganisation (more negative hub-distribution index) amongst the regulatory subnetwork in low-risk compared with high-risk subjects and SUDEP cases. * (*p* < 0.05). Error bars = SE+/−1. B, Subnetwork with highlighted regions.

All patient groups exhibited hub reorganization (as measured with HDI) within the regulatory subnetwork; this effect was entirely consistent within each subgroup (with negative slopes in all subjects). A significant main effect of group was observed for HDI (*F (2, 37)* = 5.2, *p *= 0.001). Compared with high-risk patients (*t *= 2.6, *p *= 0.014 [*D = *0.9]) and SUDEP cases (*t *= 2.7, *p *= 0.012 [*D *= 1.2]), low-risk subjects showed significantly steeper HDI slopes (greater reorganization; Fig. 5).

Lastly, GTCS frequency correlated positively with DC of the right posterior hippocampus (*r *= 0.452, *p *= 0.045), and negatively with DC of the right pregenual cingulate (*r* = −0.52, *p *= 0.02; Supplementary Figure S3A and S3B). Significant negative correlations were observed between GTCS frequency and nodal participation of the left rostroventral cingulate (*r* = −0.64, *p *= 0.002) and the right ventral caudate (*r* = −0.58, *p *= 0.008; Supplementary Figure S3C and S3D).

## 7 Discussion and conclusions

### 7.1 Overview of results

Significantly altered patterns of network connectivity emerged in SUDEP cases and living individuals at high-risk, who showed decreased network modularity and increased nodal participation relative to healthy controls amongst a subnetwork of critical brain regions involved in cardiovascular and breathing control. The findings suggest that the functional architecture among regulatory structures in SUDEP and those at greatest risk is more diffuse and less organised.

Differences in the number of connections belonging to a region (degree centrality) appeared across all patient subgroups relative to healthy controls, with SUDEP and high-risk patients eliciting similar patterns of alteration, and low-risk patients showing more drastic changes encompassing a greater number of regions. Patterns of centrality alterations, an increase in the number of hubs (hub prevalence), and reorganization of hubs among regulatory structures differentiated low-risk patients from high-risk and SUDEP cases, suggesting different pathways to functional reorganization that are related to SUDEP risk.

### 7.2 Disrupted organization among regulatory structures in SUDEP and high-risk patients

A clear division of a network into modules (assessed with modularity) is a prominent feature of many biological networks, including the mammalian cortical architecture (Yamaguti and Tsuda, 2015). Decreased modularity of resting-state functional brain networks occurs in several pathological states, e.g., childhood-onset schizophrenia (Alexander-Bloch et al., 2010), and with poorer responses to cognitive training in aging adults following traumatic brain injury (Gallen et al., 2016). Lower modularity implies a less organised network, accompanied by a reduced ability to adapt to diverse and fluctuating situations (Kashtan and Alon, 2005). We propose that aberrant organization among regulatory structures, as found here, may lead to a reduced ability of neural circuitry to adapt or respond to stimuli accompanying extreme challenges, e.g., baroreceptor, hypoxia or hypercarbia stimuli, such as might be experienced during recovery from the severe autonomic and respiratory imbalances known to accompany GTCS (Ryvlin et al., 2013; Lacuey et al., 2018; Schuele et al., 2011).

In this study nodal participation, a measure of the degree to which a brain region communicates with other modules in the regulatory subnetwork, was elevated across multiple regions in SUDEP and high-risk subjects, with the SUDEP group showing the greatest degree of changes, indicating lowered modular organization.

A possible interpretation is that higher between-module connectivity, resulting from a greater number of connector nodes (regions with higher participation), may facilitate synchrony and could enhance “channelling” of cross-network action. Such “channelling” may exaggerate neural actions, potentially overloading the system under exacerbated neuronal activity, e.g., during a GTCS, leading to unrecoverable physiological outcomes. These augmented interactions could involve brainstem regions via multiple descending influences from the diencephalon, including the thalamus. Such enhanced influences on critical nuclei in the brainstem may result in spreading depolarization(Aiba and Noebels, 2015) or disruption to brainstem networks (Mueller et al., 2014; Mueller et al., 2018).

The nature of affected areas varied across the groups. Anterior mesial temporal structures were affected, including the bilateral medial amygdala in SUDEP and bilateral anterior hippocampus in high-risk groups. These regions exert critical influences on breathing, with stimulation eliciting apnea in human epilepsy patients (Lacuey et al., 2017; Nobis et al., 2018; Dlouhy et al., 2015). Ictal central apnea is common (Lacuey et al., 2018b), prolonged instances of which may contribute to SUDEP. Increased amygdala gray matter volume has been linked with SUDEP and those at high-risk (Wandschneider, 2015). Our findings build on evidence of amygdala involvement in SUDEP and elevated risk, showing altered functional networking of these structures in high-risk and SUDEP cases.

The thalamus was, proportionally, most affected in the SUDEP group, with 10 of 16 thalamic subregions showing increased participation, implying greater inter-modular connectivity of the thalamus with other structures in the subnetwork. The bilateral medial prefrontal thalamus (mPFtha) was significantly increased in SUDEP compared with high-risk low-risk and healthy controls, and showed the greatest effect sizes, shortly followed by the posterior thalamus (Supplementary table S7). Perhaps most crucially, the medial prefrontal thalamus was increased in SUDEP compared with high-risk subjects, indicating a key difference between those at greatest risk and those who go on to succumb to SUDEP, involving the medial thalamus. Gray matter volume of the thalamus is reduced in SUDEP and those at high-risk (Wandschneider, 2015; Allen et al., 2019). Here, we demonstrate dysfunctional connectivity among many portions of the thalamus associated with SUDEP, in terms of greater reliance of these structures on other modules in the network, which was most prominent in medial and posterior aspects.

The cingulate serves critical regulatory control for blood pressure (Burns and Wyss, 1985), and interactions with other limbic structures for blood pressure control are substantial (Ongur et al., 1998). Network alterations to portions of the cingulate cortex appeared only in those who succumbed to SUDEP.

At the whole-brain level, nodal participation did not significantly differ between patient groups and controls for any region considered here. However, when exploring connectivity among the regulatory network, participation was aberrant in the patient groups. While communication between regions in the subnetwork was disrupted, these alterations went undetected when assessed in relation to the whole-brain, highlighting a well-known limitation of more-exploratory analyses, and vindicating our overarching hypothesis that connectivity amongst this subset of structures (largely forming the limbic system) is of particular relevance for SUDEP, and epilepsy.

### 7.3 Increased hub prevalence and reorganisation linked to low-risk

Degree centrality, the number of connections a node possesses to others in the regulatory subnetwork, showed increases and decreases in each patient group compared with healthy controls, with SUDEP and high-risk cases showing similar patterns. In these two groups, increases appeared in the ventromedial prefrontal cortex and right posterior hippocampus, while reductions occurred in the cingulate and insula. Low-risk patients, however, showed more widespread changes in DC, spanning a greater number of regions compared with the SUDEP and high-risk groups. Increases were principally observed in the frontal cortex (9 nodes) and decreases involved the bilateral medial thalamus, basal ganglia (5) and insula (4). Across all groups, increases appeared in the right posterior hippocampus (bilaterally in low-risk patients) and right accumbens, which may represent markers of altered connectivity specific to epilepsy in this cohort.

Additional analysis of hubs revealed that low-risk subjects demonstrated greater hub prevalence (possessed a greater number of hub nodes) among the regulatory subnetwork (despite mean connectivity) which was significantly greater than SUDEP cases. Effect sizes were sufficiently large to indicate a noteworthy increase in the number of hubs within the subnetworks of low-risk subjects compared with all other groups (Supplementary Table S9). An increase in the number of hubs demonstrated by low-risk patients may reflect improved distribution and integration of information among regions in the subnetwork (Power et al., 2013), which appears to relate to lower mortality risk. Subjects in this group did not experience GTCS, and focal seizures were sufficiently managed with anti-epileptic mediation. As such, one may speculate that the greater number of hubs, and reorganisation thereof, may reflect to improved functional brain networking in people with more successfully controlled epilepsy.

The hub distribution index, the extent to which hubs (highly connected nodes) are topologically organised in patients with respect to the control group, was negative in all patients, reflecting a shift in the organization of functional brain network hubs within the subnetwork; the same is apparent in comatose individuals (Achard et al., 2012). Moreover, HDI slopes were significantly steeper (more negative) in low-risk patients, when compared with SUDEP and high-risk patients. This change indicates a dramatic shift in hub organization within the regulatory subnetwork in low-risk patients. The same reorganization in low-risk patients did not appear when computed for the whole-brain.

Taken together, the alterations in DC, elevated hub prevalence and greater hub reorganization differentiated patients at low-risk, and may provide evidence of network reorganization among regulatory sites in subjects with better-controlled epilepsy. Imaging features related to reduced SUDEP risk may provide further insight into mechanisms and prevention approaches; thus, these findings warrant further investigation, as they may one day have implications for neuromodulatory interventions.

### 7.4 GTCS frequency and graph measures

When considered over the subnetwork of interest only, GTCS frequency correlated negatively with participation in the left rostroventral anterior cingulate, a region which increased participation in SUDEP cases. None of the subnetwork regions showing increased participation in high-risk and SUDEP cases demonstrated significant positive correlations with GTCS frequency. The only significant negative correlation between GTCS frequency and DC was in the right pregenual cingulate, suggesting that repeated GTCS are related to reduced connectivity of this region, a concern, since stimulation near here leads to central apnea (Lacuey et al., 2018). Conversely, the only positive correlation between DC and GTCS frequency appeared in the right posterior hippocampus, suggesting more frequent GTCS are related to greater connectivity of this structure. GTCS frequency did not significantly correlate with modularity or hub distribution across the subnetwork. Overall, GTCS frequency is an important SUDEP risk factor and correlates with connectivity measures of several regions. However, GTCS seizure frequency alone could not explain the drastic alterations in modularity and participation observed in cases of SUDEP or high-risk patients.

### 7.5 Autonomic dysregulation in epilepsy and functional imaging biomarkers of SUDEP

A meta-analysis of heart rate variability in epilepsy (Lotufo et al., 2012) revealed sympathovagal imbalance, with lower vagal and higher sympathetic tone in patients. High sympathetic tone poses a risk, leading to constriction of the arterial supply to vital organs, and to arrhythmia, especially if the outflow is asymmetric (Schwartz, Priori, and Napolitano 2000). Conversely, while routine vagal activity levels are often cardioprotective, excessive activity leads to bradycardia and impaired perfusion (Thayer et al., 2010). The disrupted connectivity in key cardiovascular and breathing sites, including the amygdala, hippocampus and thalamus (Frysinger and Harper, 1989; Macey et al., 2016), in SUDEP and high-risk groups may provide insights into mechanisms of central autonomic dysfunction related to SUDEP.

Connectivity of mesial temporal (hippocampus and amygdala) and thalamic structures was extensively altered in SUDEP and in high-risk patients. We, and others, previously found that volumetric and functional connectivity changes occur in the hippocampus, amygdala and thalamus in SUDEP and those at high-risk (Wandschneider, 2015; Ogren et al., 2018; Tang et al., 2014; Allen et al., 2017, Allen et al., 2019), findings which reinforce the importance of these structures to SUDEP and elevated risk. In this respect, a major finding of the present study was significantly altered connectivity of the medial prefrontal thalamus in SUDEP when compared with all other sub-groups. Altered connectivity of this structure indicates a potential non-invasive, functional connectivity-based, marker of elevated SUDEP risk.

### 7.6 Limitations and future work

The difficulty of identifying SUDEP cases makes imaging studies challenging, usually resulting in small sample sizes; a limitation of the current study. If future studies (particularly those employing novel imaging sequences such as rs-fMRI) are to expand their sample sizes, large multi-center collaborations should be sought and methodological issues surrounding multiple MRI scanners will require careful consideration.

The time between scanning and death of SUDEP cases is a caveat of the current study, not least since this time interval varied across cases (between 2 and 8 years, mean = 4.9 ± 2.2), but also as the changes observed in the current dataset reflect connectivity alterations which were characterised may years prior to the occurrence of SUDEP. However, given the rarity of the current data, this caveat should be appreciated in the context of the challenges faced in obtaining such cases.

Methodological constraints precluded inclusion of the brainstem and cerebellum here, despite their central roles in autonomic and respiratory regulation. Typically, resting-state fMRI scans employing graph theory do not cover the brainstem region due to noise introduced from non-gray matter signals (Brooks et al., 2013). Future studies should also explore brainstem connectivity using diffusion MRI, which would allow assessment of white matter tracts in the region that are difficult to investigate with fMRI. Insufficient imaging coverage of the cerebellum in many subjects precluded its inclusion in this analysis.

Although our selection of regulatory sub-network nodes may be seen as somewhat arbitrary, extensive previous work demonstrated the regional roles in autonomic and respiratory control (Macey et al., 2016; Shoemaker and Goswami, 2015; Loewy, 1991), and earlier studies constructed similar (but less detailed) subnetworks (Allen et al., 2017). Future studies, however, should record autonomic and respiratory output (i.e. blood pressure, breathing rate, apnea) during fMRI for inclusion in connectivity analyses.

We selected the brainnetome atlas due to its structurally and functionally relevant sub-divisions, of which there are many more than any other existing template atlas available for use in fMRI analysis. In addition to this, we performed modularity analysis on the whole brain and a subnetwork, based on a different atlas, but albeit one with fewer sub-divisions – the Harvard-Oxford (HO) cortical and sub-cortical atlas. The HO includes 112 brain regions, from which we built a subnetwork of regulatory ROIs consisting of 32 regions (see Supplementary table S10 for details). The same pattern of reduced modularity of the subnetwork in patients was reproduced using the H&O atlas (Supplementary Figure S4), with high-risk and SUDEP showing large reductions in modularity compared with healthy controls. However, these did not reach significance: low-risk (*t *= 0.54, *p *= 0.61 [*D *= 0.18]), high-risk (*t *= 1.54, *p *= 0.13.[*D *= 0.56]), SUDEP (*t *= 1.83, *p *= 0.7 [*D *= 0.80]). It is possible that which such fewer nodes (half compared with the subnetwork constructed from the brainnetome), and thus a network of lower resolution, less information is captured by graph theoretical measurements. Although this requires further work, a potential advantage of constructing networks based on parcellations with more subdivisions is highlighted, since a greater number of nodes may enable greater network complexity and improved anatomical and functional specificity of networks to be captured.

Matching high-risk subjects to SUDEP cases based on clinical variables, such as epilepsy duration and seizure frequency, enabled us to account for epilepsy severity (a confound and popular critique of SUDEP studies). Doing so ensures that the main difference between these two groups is that the former were alive at the time of inclusion and the latter were not due to SUDEP. Interestingly, SUDEP and high-risk showed similar connectivity alterations compared with healthy controls. However, the differences between them are of note. For example, although modularity was reduced in both SUDEP and high-risk, the magnitude of difference was greater in SUDEP. Also, nodal participation was elevated in both SUDEP and high-risk, although the number and nature of affected nodes differed (SUDEP showed increases among 23 regions, involving mainly thalamic nodes, while high-risk exhibited increases among 16 nodes, with mainly insula nodes affected). Lastly, and importantly, a key difference in nodal participation emerged between SUDEP and high-risk – increases in the bilateral medial prefrontal thalamus and the posterior thalamus. Further investigation into these patterns may be key to understanding the processes leading to SUDEP.

## 8 Conclusions

The functional organization among regions involved in respiratory and cardiovascular regulation was less modular in patients who subsequently succumbed to SUDEP and in living subjects at high-risk. Disrupted organization could result in impaired communication among regulatory sites, particularly under extreme scenarios. Greater inter-modular connectivity may reflect an increased propensity to facilitate seizure spread and promote excessive neuronal interactions among vital structures. Increases in the number, and a shift in organisation, of hubs, found in those at low-risk, may reflect improved distribution and integration of information among regulatory regions, which appears to relate to lower mortality risk. The characterization of altered network properties among essential autonomic and breathing regulatory brain areas may shed light on the processes underlying SUDEP and facilitate non-invasive evaluation of SUDEP risk stratification.

## Appendix Supplementary materials

Image, application 1
