# Supplementary material for: Altered brain connectivity in sudden unexpected death in epilepsy (SUDEP) revealed using resting-state fMRI
Source: Neuroimage Clin. 2019 Oct 28;24:102060. doi: 10.1016/j.nicl.2019.102060 (PMC6849487; doi:10.1016/j.nicl.2019.102060)
Supplement: Supplementary file 1 [file mmc1.docx]

1. **Supplementary tables**

**Table S1.** Clinical characteristics of low- and high-risk patient- and healthy-control subjects. M=male, F=female, L=left, R=right, JME=juvenile myoclonic epilepsy, FCD=focal cortical dysplasia, MFG=middle frontal gyrus, IFG=inferior frontal gyrus, STG=superior temporal gyrus, hem=hemisphere, HS=hippocampal sclerosis, HC=healthy control, N.A.=not applicable.

| **Case # [sex]** | **Risk group** | **Age at time of scan**  **(years)** | **Epilepsy syndrome** | **Disease duration**  **(years)** | **Number of GTCS**  **(per month)** | **MRI findings at time of scan** |
| --- | --- | --- | --- | --- | --- | --- |
| 1 [M] | High-risk | 20 | Focal, L frontal | 9 | 2 | Normal |
| 2 [M] | High-risk | 25 | Focal, L frontal | 15 | 3 | L MFG FCD |
| 3 [M] | High-risk | 33 | Generalised (JME) | 20 | 1 | Normal |
| 4 [M] | High-risk | 35 | Generalised | 29 | 2 | Normal |
| 5 [F] | High-risk | 18 | Generalised (JME) | 15 | 2.5 | Normal |
| 6 [F] | High-risk | 26 | Generalised (JME) | 24 | 1.5 | Normal |
| 7 [M] | High-risk | 49 | Focal, L frontal | 45 | 3 | Normal |
| 8 [M] | High-risk | 23 | Focal, L fronto-temporal | 11 | 1 | Normal |
| 9 [F] | High-risk | 37 | Focal, L frontal | 21 | 1.5 | Normal |
| 10 [F] | High-risk | 46 | Focal, L frontal | 41 | 10 | L IFG FCD |
| 11 [F] | High-risk | 19 | Focal, R parietal | 17 | 2 | L parietal FCD |
| 12 [F] | High-risk | 25 | Focal, R parietal | 24 | 5 | L parietal FCD |
| 13 [M] | High-risk | 22 | Focal, L temporal | 18 | 8 | L HS |
| 14 [M] | High-risk | 33 | Focal, L temporal | 31 | 6.5 | L HS |
| 15 [F] | High-risk | 31 | Focal, L fronto-temporal | 25 | 8 | Normal |
| 16 [F] | High-risk | 26 | Focal, L fronto-temporal | 17 | 10 | Normal |
| 17 [M] | Low-risk | 24 | Focal, L frontal | 17 | 0 | Normal |
| 18 [M] | Low-risk | 24 | Focal, L frontal | 20 | 0 | L frontal FCD |
| 19 [M] | Low-risk | 39 | Generalised | 35 | 0 | Normal |
| 20 [M] | Low-risk | 33 | Generalised | 17 | 0 | Normal |
| 21 [F] | Low-risk | 30 | Generalised (JME) | 17 | 0 | Normal |
| 22 [F] | Low-risk | 38 | Generalised | 31 | 0 | Normal |
| 23 [M] | Low-risk | 27 | Focal, L Parietal | 20 | 0 | L parietal FCD |
| 24 [M] | Low-risk | 33 | Focal, L hem | 18 | 0 | Normal |
| 25 [F] | Low-risk | 24 | Focal, L frontal | 21 | 0 | Normal |
| 26 [F] | Low-risk | 42 | Focal, L frontal | 24 | 0 | L frontal FCD |
| 27 [F] | Low-risk | 28 | Focal, R parieto-occipital | 21 | 0 | R parieto-occipital FCD |
| 28 [F] | Low-risk | 32 | Focal, R parietal | 30 | 0 | R superior parietal FCD |
| 29 [M] | Low-risk | 26 | Focal, L temporal | 19 | 0 | L HS |
| 30 [M] | Low-risk | 28 | Focal, L temporal | 23 | 0 | L STG FCD |
| 31 [F] | Low-risk | 19 | Focal, L frontal | 13 | 0 | L inferior frontal infarct |
| 32 [F] | Low-risk | 24 | Focal, L frontal | 20 | 0 | Normal |
| 33 [F] | HC | 30 | N.A. | N.A. | N.A. | N.A. |
| 34 [M] | HC | 30 | N.A. | N.A. | N.A. | N.A. |
| 35 [F] | HC | 49 | N.A. | N.A. | N.A. | N.A. |
| 36 [M] | HC | 22 | N.A. | N.A. | N.A. | N.A. |
| 37 [M] | HC | 28 | N.A. | N.A. | N.A. | N.A. |
| 38 [M] | HC | 28 | N.A. | N.A. | N.A. | N.A. |
| 39 [F] | HC | 29 | N.A. | N.A. | N.A. | N.A. |
| 40 [F] | HC | 25 | N.A. | N.A. | N.A. | N.A. |
| 41 [M] | HC | 35 | N.A. | N.A. | N.A. | N.A. |
| 42 [F] | HC | 27 | N.A. | N.A. | N.A. | N.A. |
| 43 [M] | HC | 34 | N.A. | N.A. | N.A. | N.A. |
| 44 [M] | HC | 30 | N.A. | N.A. | N.A. | N.A. |
| 45 [F] | HC | 33 | N.A. | N.A. | N.A. | N.A. |
| 46 [F] | HC | 28 | N.A. | N.A. | N.A. | N.A. |
| 47 [M] | HC | 28 | N.A. | N.A. | N.A. | N.A. |
| 48 [M] | HC | 33 | N.A. | N.A. | N.A. | N.A. |

**Table S2.** Group summaries of SUDEP cases, high-risk, low-risk and healthy controls. SD=standard deviation, M=male, F=female, /=per, AED=anti-epileptic drug, HC=healthy control, N.A.=not applicable.

| **Variable** | **SUDEP (n=8)** | **High-risk (n=16)** | **Low-risk (n=16)** | **HC (n=16)** |
| --- | --- | --- | --- | --- |
| Age (mean±SD) | 26 ± 6.1 | 29.3 ± 9.2 | 29.4 ± 6.3 | 30.6 ± 6 |
| Gender (M:F) | 4:4 | 8:8 | 8:8 | 9:7 |
| Disease duration (years) | 16.6 ± 10.1 | 22.6 ± 10.0 | 21.6 ± 5.8 | N.A. |
| GTCS/month (mean±SD) | 3.0 ±3.1 | 5.4 ± 7.1 | N.A. | N.A. |
| Number of AEDs (mean±SD) | 2.6 ± 0.5 | 2.6 ± 0.9 | 2.8 ± 0.9 | N.A. |
| Number polytherapy | 5 | 8 | 9 | N.A. |
| Number duotherapy | 3 | 7 | 7 | N.A. |
| Number monotherapy | N.A. | 1 | N.A. | N.A. |

**Table S3.** Brainnetome atlas regions, labels, abbreviations and MNI co-ordinates. Abbrev = abbreviation.

| **Structure** | **Sub-region label** | **Abbrev** | **MNI Left**  **(X, Y, Z)** | **MNI Right**  **(X, Y, Z)** |
| --- | --- | --- | --- | --- |
| Medial/orbital-frontal cortex | Medial prefrontal (anterior 1) | aMpf1 | -7, 54, -7 | 6, 47, -7 |
|  | Orbital (ventrolateral 1) | vlOrb1 | -36, 33, -16 | 40, 39, -14 |
|  | Orbital (ventrolateral 2) | vlOrb2 | -23, 38, -18 | 23, 36, -18 |
|  | Medial prefrontal (anterior 2) | aMpf2 | -6, 52, -19 | 6, 57, -16 |
|  | Ventromedial prefrontal | vMpf | -10, 18, -19 | 9, 20, -19 |
|  | Orbital (dorsolateral) | dlOrb | -41, 32, -9 | 42, 31, -9 |
| Insula | hypergranular ins | Hg_ins | -36, -20, 10 | 37, -18, 8 |
|  | ventral agranular ins | vAng_ins | -32, 14, -13 | 33, 14, -13 |
|  | dorsal agranular ins | dAng_ins | -34, 18, 1 | 36, 18, 1 |
|  | ventral dysgranular+granular ins | vD&G_ins | -38, -4, -9 | 39, -2, -9 |
|  | dorsal granular ins | dGran_ins | -38, -8, 8 | 39, -7, 8 |
|  | dorsal dysgranular ins | dDysg_ins | -38, 5, 5 | 38, 5, 5 |
| Cingulate | dorsal area | dCing | -4, -39, 31 | 4, -37, 32 |
|  | rostroventral area | rvCing | -3, 8, 25 | 5, 22, 12 |
|  | pregenual area | Pregen | -6, 34, 21 | 5, 28, 27 |
|  | ventral area | vCing | -8, -47, 10 | 9, -44, 11 |
|  | caudodorsal area | cd_Cing | -5, 7, 37 | 4, 6, 38 |
|  | caudal area | cCing | -7, -23, 41 | 6, -20, 40 |
|  | subgenual area | Subgen | -4, 39, -2 | 5, 41, 6 |
| Amygdala | medial amygdala | mAmyg | -19, -2, -20 | 19, -2, -19 |
|  | lateral amygdala | lAmyg | -27, -4, -20 | 28, -3, -20 |
| Hippocampus | rostral hippocampus | aHipp | -22, -14, -19 | 22, -12, -20 |
|  | caudal hippocampus | pHipp | -28, -30, -10 | 29, -27, -10 |
| Basal Ganglia | ventral caudate | vCaud | -12, 14, 0 | 15, 14, -2 |
|  | globus pallidus | GP | -22, -2, 4 | 22, -2, 3 |
|  | NAC, nucleus accumbens | NAC | -17, 3, -9 | 15, 8, -9 |
|  | ventromedial putamen | vmPut | -23, 7, -4 | 22, 8, -1 |
|  | dorsal caudate | dCaud | -14, 2, 16 | 14, 5, 14 |
|  | dorsolateral putamen | dlPut | -28, -5, 2 | 29, -3, 1 |
| Thalamus | medial pre-frontal thalamus | mPFtha | -7, -12, 5 | 7, -11, 6 |
|  | pre-motor thalamus | mPMtha | -18, -13, 3 | 12, -14, 1 |
|  | sensory thalamus | Stha | -18, -23, 4 | 18, -22, 3 |
|  | rostral temporal thalamus | rTtha | -7, -14, 7 | 3, -13, 5 |
|  | posterior parietal thalamus | Pptha | -16, -24, 6 | 15, -25, 6 |
|  | occipital thalamus | Octha | -15, -28, 4 | 13, -27, 8 |
|  | caudal temporal thalamus | cTtha | -12, -22, 13 | 10, -14, 14 |
|  | lateral pre-frontal thalamus | lPFtha | -11, -14, 2 | 13, -16, 7 |

1. **Supplementary methods**

**i. *RS-fMRI* Data Processing**

***a) Pre-processing***

The software package ‘Data Processing Assistant for Resting-State fMRI’ (DPARSFA; Yan & Zang, 2010), running in Matlab 2017b (MathWorks, USA), was used to pre-process the fMRI data. DPARSFA utilises functions from the software packages REST (Resting-State fMRI Data Analysis Toolkit; Song et al., 2011) and SPM12 (Statistical Parametric Mapping; www.fil.ion.ucl.ac.uk/spm). For each subject, the high-resolution T1 image was segmented using a fast-diffeomorphic image registration algorithm (DARTEL; Ashburner, 2007). The functional MRI volumes were slice timing-corrected and realigned. The 6-motion realignment parameters (generated during functional image realignment), together with the mean white matter and CSF (generated during segmentation), were removed during nuisance covariate regression. Further motion correction (or ‘scrubbing’) was implemented to account for subtle (sub-millimetre) head movements which confound rs-fMRI data despite routine motion correction (Power et al., 2012). This correction was achieved by calculating the frame-wise displacement (FD – the sum of the absolute values of the realignment estimates relative to the preceding scan; Power et al., 2012), and replacing the volumes for which FD exceeded 0.5mm with linearly interpolated values. A threshold of 0.5mm was selected as a trade-off between appropriate control over movement and a reasonable amount of un-corrected data remaining per subject (in our data, no more than 30% of the volumes were corrected for a given subject). Lastly, the Brainnetome (BNA) atlas (Fan et al, 2016) was warped into individual scan space, using the parameters generated by DARTEL, for rs-fMRI time series extraction.

***b) Wavelet filtering***

Prior to wavelet filtering, all time-series were shortened to 200 volumes (approximately 10 mins), due to 5 subjects having undergone a shortened scan. The average fMRI time series was calculated over all voxels in each of the 246 regions of the Brainnetome atlas and decomposed using the maximal overlap discrete wavelet transform (MODWT; Percival & Walden, 2000). We restricted our analysis to scale 2 of the wavelet decomposition which, in our data, corresponded to the frequency range 0.03~0.06Hz, the range at which small-world network properties are most salient (Achard et al., 2006; Bullmore et al., 2004). Wavelet filtering was carried out using the Matlab Wavelet Toolbox.

**ii. Network Construction and Analysis**

The wavelet coefficients obtained from the steps described above were used to perform inter-regional correlations between every brain region (or “node”), generating for each subject a 74×74 weighted network (or “graph”) for the regulatory subnetwork, and a 246×246 weighted network for the whole brain. Network construction and computation of the network measures were carried out using the Brain Connectivity Toolbox (BCT; Rubinov & Sporns, 2010) in Matlab. Weighted networks were thresholded using a minimal spanning tree (MST) approach, whereby the lowest weighted “edges” (connections between nodes) are used to minimally connect the network (Kruskal, 1956; Prim, 1957). Connections were then added back to the MST in descending order of the wavelet coefficients at network densities (proportion of connections) ranging from 50% to 5% in decrements of 1% (Alexander-Bloch et al, 2010). This procedure yielded a series of 46 binary undirected (involving non-directional connections) networks per subject, on which the four following measures were computed (the equations of which can be found in Section 2a below).

In the following we summarise the mathematical formalism behind the network metrics used in this work, as implemented in BCT.

1. **Network Modularity**

Modularity was computed using the Louvain method for community detection (Blondel et al., 2008), which utilises a multi-iterative algorithm to determine the best possible moduli of nodes into groups and thus an optimised (or ‘maximised’) modularity. In summary, modularity was estimated and optimised through a two-phase iterative process (Blondel et al., 2008), and was initially defined as:

$$Q=\frac{1}{2m}\sum_{ij} \left[ A_{ij}-\frac{k_{i} k_{j}}{2m} \right]\delta\left( c_{i},c_{j} \right)$$

Where $A_{ij}$ represents the edge weight between nodes $i$ and $j$; for binary networks, as in the current instance, this value would be 0 or 1; $k_{i}$ and $k_{j}$ are the sum of the number of edges attached to nodes $i$ and $j$ respectively; $2m$ is the sum of all edges in the graph; $c_{i}$ and $c_{j}$ are the communities of the nodes; and $\delta$ is a delta function.

In order for this value to be efficiently optimised, two phases are repeated iteratively. First, each node in the network is assigned to its own community. Then for each node $i$, the change in modularity is calculated for removing $i$ from its own community and moving it into the community of each neighbour $j$ of $i$. This value is calculated by: (1) removing $i$ from its original community, and (2) inserting $i$ to the community of $j$. The equation for step 2 is as follows:

$$\Delta Q=\left[ \frac{\Sigma_{in}+2k_{i,in}}{2m}-\left( \frac{\Sigma_{tot}+k_{i}}{2m} \right)^{2} \right]-\left[ \frac{\Sigma_{in}}{2m}-\left( \frac{\Sigma_{tot}}{2m} \right)^{2}-\left( \frac{k_{i}}{2m} \right)^{2} \right]$$

Where $\Sigma_{in}$ is sum of all links inside the community $i$ is moving into, $\Sigma_{tot}$ is the sum of all links to nodes in the community $i$ is moving into, $k_{i}$ is the degree of $i$, $k_{i,in}$ is the sum of the links between $i$ and other nodes in the community that $i$ is moving into, and $m$ is the sum of all links in the network. Once this value is calculated for all communities to which $i$ is connected, $i$ is placed into the community that resulted in the greatest increase in modularity. If no increase is possible, $i$ remains in its original community. This process is carried out repeatedly and sequentially to all nodes until no modularity increase can occur.

The first phase is over once the local maximum of modularity is found. In the second phase, the nodes in the same community are grouped and a new network is built where nodes are the communities from the previous phase. Any links between nodes of the same community are now represented by self-loops on the new community node, and links from multiple nodes in the same community to a node in a different community are represented by links between communities. Once the new network is created, the second phase is over and phase 1 can be re-applied to the new network.

1. **Nodal Participation**

The participation coefficient (Guimera And Amaral, 2005) is a measure of the diversity of intermodular interconnections of a given node in the network,^11^ and is defined as:

$$y_{i}=1-\sum_{m\epsilon M} \left( \frac{k_{i}}{2m} \right)^{2}$$

Where $M$ is the set of modules and $k_{i}$ $(m)$ is the number of link between $i$ and all nodes in module $M$.

1. **Nodal Degree Centrality (DC)**

DC is simply defined as the number of connections incident upon a node after thresholding (Rubinov & Sporns, 2010).

**3. Supplementary results tables**

**Table S4.** Increased regional participation in SUDEP (compared with high-risk, low-risk and healthy controls) and high-risk groups (compared with healthy controls only). P-values from two-sample permutation tests are reported, and ones below 0.05 are highlighted in red (FDR corrected) and yellow (uncorrected).

| **Structure** | **Sub-region** | **SUDEP > HC**  **(FDR corrected *p-v*alue)** | **High-risk > HC**  **(FDR corrected *p*-value)** | **SUDEP > low-risk**  **(uncorrected *p*-value)** | **SUDEP > high-risk**  **(uncorrected *p*-value)** |
| --- | --- | --- | --- | --- | --- |
| Medial/orbital-frontal cortex | aMpf1_L | 0.198 | 0.144 | 0.223 | 0.462 |
|  | aMpf1_R | 0.288 | 0.095 | 0.481 | 0.710 |
|  | vlOrb1_L | 0.085 | 0.114 | 0.161 | 0.294 |
|  | vlOrb1_R | 0.288 | 0.144 | 0.527 | 0.662 |
|  | vlOrb2_L | 0.038 | 0.044 | 0.251 | 0.266 |
|  | vlOrb2_R | 0.150 | 0.039 | 0.429 | 0.832 |
|  | aMpf2_L | 0.110 | 0.079 | 0.397 | 0.377 |
|  | aMpf2_R | 0.358 | 0.091 | 0.534 | 0.764 |
|  | vMpf_L | 0.103 | 0.140 | 0.479 | 0.267 |
|  | vMpf_R | 0.060 | 0.119 | 0.167 | 0.311 |
|  | dlOrb_L | 0.035 | 0.126 | 0.107 | 0.090 |
|  | dlOrb_R | 0.361 | 0.044 | 0.543 | 0.903 |
| Insula | Hg_ins_L | 0.038 | 0.044 | 0.286 | 0.419 |
|  | Hg_ins_R | 0.362 | 0.218 | 0.807 | 0.818 |
|  | vAng_ins_L | 0.028 | 0.024 | 0.341 | 0.603 |
|  | vAng_ins_R | 0.288 | 0.126 | 0.718 | 0.672 |
|  | dAng_ins_L | 0.193 | 0.044 | 0.609 | 0.778 |
|  | dAng_ins_R | 0.097 | 0.061 | 0.312 | 0.394 |
|  | vD&G_ins_L | 0.129 | 0.039 | 0.814 | 0.849 |
|  | vD&G_ins_R | 0.064 | 0.052 | 0.470 | 0.413 |
|  | dGran_ins_L | 0.080 | 0.024 | 0.655 | 0.863 |
|  | dGran_ins_R | 0.038 | 0.039 | 0.157 | 0.560 |
|  | dDysg_ins_L | 0.288 | 0.024 | 0.828 | 0.899 |
|  | dDysg_ins_R | 0.129 | 0.052 | 0.406 | 0.817 |
| Cingulate | dCing_L | 0.164 | 0.119 | 0.151 | 0.315 |
|  | dCing_R | 0.187 | 0.111 | 0.282 | 0.555 |
|  | rvCing_L | 0.063 | 0.065 | 0.326 | 0.291 |
|  | rvCing_R | 0.131 | 0.059 | 0.758 | 0.675 |
|  | Pregen_L | 0.077 | 0.052 | 0.450 | 0.404 |
|  | Pregen_R | 0.097 | 0.358 | 0.190 | 0.149 |
|  | vCing_L | 0.251 | 0.230 | 0.258 | 0.514 |
|  | vCing_R | 0.085 | 0.252 | 0.453 | 0.382 |
|  | cd_Cing_L | 0.080 | 0.144 | 0.400 | 0.461 |
|  | cd_Cing_R | 0.086 | 0.080 | 0.371 | 0.415 |
|  | cCing_L | 0.288 | 0.144 | 0.663 | 0.697 |
|  | cCing_R | 0.035 | 0.132 | 0.504 | 0.135 |
|  | Subgen_L | 0.043 | 0.091 | 0.037 | 0.308 |
|  | Subgen_R | 0.077 | 0.137 | 0.437 | 0.379 |
| Amygdala | mAmyg_L | 0.005 | 0.052 | 0.061 | 0.154 |
|  | mAmyg_R | 0.033 | 0.093 | 0.228 | 0.418 |
|  | lAmyg_L | 0.028 | 0.144 | 0.170 | 0.081 |
|  | lAmyg_R | 0.358 | 0.618 | 0.782 | 0.351 |
| Hippocampus | aHipp_L | 0.080 | 0.039 | 0.324 | 0.567 |
|  | aHipp_R | 0.039 | 0.039 | 0.111 | 0.239 |
|  | pHipp_L | 0.077 | 0.182 | 0.118 | 0.073 |
|  | pHipp_R | 0.015 | 0.040 | 0.031 | 0.065 |
| Basal Ganglia | vCaud_L | 0.322 | 0.097 | 0.509 | 0.800 |
|  | vCaud_R | 0.164 | 0.343 | 0.023 | 0.260 |
|  | GP_L | 0.288 | 0.272 | 0.623 | 0.450 |
|  | GP_R | 0.074 | 0.097 | 0.075 | 0.250 |
|  | NAC_L | 0.138 | 0.091 | 0.227 | 0.626 |
|  | NAC_R | 0.080 | 0.052 | 0.143 | 0.504 |
|  | vmPut_L | 0.080 | 0.080 | 0.143 | 0.356 |
|  | vmPut_R | 0.030 | 0.119 | 0.050 | 0.201 |
|  | dCaud_L | 0.430 | 0.243 | 0.592 | 0.629 |
|  | dCaud_R | 0.223 | 0.024 | 0.781 | 0.906 |
|  | dlPut_L | 0.361 | 0.097 | 0.575 | 0.813 |
|  | dlPut_R | 0.288 | 0.144 | 0.769 | 0.675 |
| Thalamus | mPFtha_L | 0.007 | 0.146 | 0.027 | 0.038 |
|  | mPFtha_R | 0.006 | 0.144 | 0.004 | <0.001 |
|  | mPMtha_L | 0.059 | 0.052 | 0.048 | 0.288 |
|  | mPMtha_R | 0.147 | 0.097 | 0.619 | 0.428 |
|  | Stha_L | 0.035 | 0.097 | 0.456 | 0.145 |
|  | Stha_R | 0.063 | 0.272 | 0.584 | 0.089 |
|  | rTtha_L | 0.308 | 0.067 | 0.775 | 0.860 |
|  | rTtha_R | 0.033 | 0.093 | 0.095 | 0.030 |
|  | Pptha_L | 0.038 | 0.039 | 0.327 | 0.457 |
|  | Pptha_R | 0.007 | 0.209 | 0.081 | 0.013 |
|  | Octha_L | 0.021 | 0.040 | 0.108 | 0.160 |
|  | Octha_R | 0.021 | 0.091 | 0.044 | 0.155 |
|  | cTtha_L | 0.033 | 0.108 | 0.441 | 0.098 |
|  | cTtha_R | 0.036 | 0.141 | 0.312 | 0.203 |
|  | lPFtha_L | 0.201 | 0.126 | 0.649 | 0.615 |
|  | lPFtha_R | 0.069 | 0.163 | 0.218 | 0.158 |

**Table S5.** Increased degree centrality (DC) in SUDEP, high-risk and low-risk compared with healthy controls, assessed with two-sample permutation t-tests. FDR corrected p-values are displayed and those below 0.05 are highlighted in red.

| **Structure** | **Sub-region** | **SUDEP > HC (*p-v*alue)** | **High-risk > HC (p-value)** | **Low-risk > HC (p-value)** |
| --- | --- | --- | --- | --- |
| Medial/orbital-frontal cortex | aMpf1_L | 0.164 | 0.751 | 0.574 |
|  | aMpf1_R | 0.508 | 0.877 | 0.341 |
|  | vlOrb1_L | 0.135 | 0.064 | 0.003 |
|  | vlOrb1_R | 0.038 | 0.002 | 0.007 |
|  | vlOrb2_L | 0.187 | 0.247 | 0.005 |
|  | vlOrb2_R | 0.096 | 0.096 | 0.023 |
|  | aMpf2_L | 0.083 | 0.234 | 0.030 |
|  | aMpf2_R | 0.008 | 0.261 | 0.028 |
|  | vMpf_L | 0.028 | 0.010 | 0.002 |
|  | vMpf_R | 0.009 | 0.013 | 0.026 |
|  | dlOrb_L | 0.335 | 0.476 | 0.034 |
|  | dlOrb_R | 0.464 | 0.520 | 0.508 |
| Insula | Hg_ins_L | 0.864 | 0.878 | 0.471 |
|  | Hg_ins_R | 0.985 | 0.885 | 0.983 |
|  | vAng_ins_L | 0.127 | 0.184 | 0.143 |
|  | vAng_ins_R | 0.321 | 0.537 | 0.464 |
|  | dAng_ins_L | 0.939 | 0.719 | 0.482 |
|  | dAng_ins_R | 0.675 | 0.798 | 0.993 |
|  | vD&G_ins_L | 0.765 | 0.682 | 0.451 |
|  | vD&G_ins_R | 0.959 | 0.988 | 0.854 |
|  | dGran_ins_L | 0.671 | 0.847 | 0.649 |
|  | dGran_ins_R | 0.833 | 0.755 | 0.994 |
|  | dDysg_ins_L | 0.828 | 0.541 | 0.450 |
|  | dDysg_ins_R | 0.884 | 0.385 | 0.959 |
| Cingulate | dCing_L | 0.148 | 0.687 | 0.140 |
|  | dCing_R | 0.174 | 0.577 | 0.526 |
|  | rvCing_L | 0.061 | 0.030 | 0.109 |
|  | rvCing_R | 0.491 | 0.250 | 0.139 |
|  | Pregen_L | 0.784 | 0.751 | 0.826 |
|  | Pregen_R | 0.290 | 0.977 | 0.945 |
|  | vCing_L | 0.185 | 0.170 | 0.073 |
|  | vCing_R | 0.277 | 0.166 | 0.064 |
|  | cd_Cing_L | 0.818 | 0.766 | 0.762 |
|  | cd_Cing_R | 0.334 | 0.623 | 0.507 |
|  | cCing_L | 0.998 | 0.998 | 0.687 |
|  | cCing_R | 0.893 | 0.990 | 0.655 |
|  | Subgen_L | 0.404 | 0.865 | 0.660 |
|  | Subgen_R | 0.435 | 0.891 | 0.826 |
| Amygdala | mAmyg_L | 0.080 | 0.284 | 0.108 |
|  | mAmyg_R | 0.098 | 0.331 | 0.544 |
|  | lAmyg_L | 0.467 | 0.459 | 0.351 |
|  | lAmyg_R | 0.817 | 0.465 | 0.726 |
| Hippocampus | aHipp_L | 0.510 | 0.187 | 0.134 |
|  | aHipp_R | 0.176 | 0.085 | 0.156 |
|  | pHipp_L | 0.302 | 0.061 | 0.036 |
|  | pHipp_R | 0.003 | 0.018 | 0.036 |
| Basal Ganglia | vCaud_L | 0.401 | 0.694 | 0.603 |
|  | vCaud_R | 0.554 | 0.750 | 0.895 |
|  | GP_L | 0.895 | 0.828 | 0.950 |
|  | GP_R | 0.525 | 0.687 | 0.965 |
|  | NAC_L | 0.723 | 0.639 | 0.902 |
|  | NAC_R | 0.025 | 0.010 | 0.036 |
|  | vmPut_L | 0.870 | 0.945 | 0.937 |
|  | vmPut_R | 0.765 | 0.794 | 0.960 |
|  | dCaud_L | 0.595 | 0.750 | 0.686 |
|  | dCaud_R | 0.396 | 0.189 | 0.167 |
|  | dlPut_L | 0.948 | 0.701 | 0.955 |
|  | dlPut_R | 0.920 | 0.941 | 0.971 |
| Thalamus | mPFtha_L | 0.561 | 0.588 | 0.984 |
|  | mPFtha_R | 0.197 | 0.780 | 0.982 |
|  | mPMtha_L | 0.177 | 0.094 | 0.592 |
|  | mPMtha_R | 0.967 | 0.796 | 0.975 |
|  | Stha_L | 0.695 | 0.402 | 0.941 |
|  | Stha_R | 0.824 | 0.238 | 0.916 |
|  | rTtha_L | 0.835 | 0.247 | 0.697 |
|  | rTtha_R | 0.885 | 0.291 | 0.862 |
|  | Pptha_L | 0.962 | 0.258 | 0.852 |
|  | Pptha_R | 0.735 | 0.767 | 0.569 |
|  | Octha_L | 0.187 | 0.445 | 0.396 |
|  | Octha_R | 0.322 | 0.351 | 0.307 |
|  | cTtha_L | 0.745 | 0.558 | 0.604 |
|  | cTtha_R | 0.564 | 0.552 | 0.089 |
|  | lPFtha_L | 0.978 | 0.803 | 0.872 |
|  | lPFtha_R | 0.925 | 0.972 | 0.944 |

**Table S6.** Degree centrality in SUDEP, high-risk and low-risk compared with healthy controls. FDR corrected p-values are displayed and those below 0.05 are highlighted in green.

| **Structure** | **Sub-region** | **SUDEP < HC (*p-v*alue)** | **High-risk < HC (p-value)** | **Low-risk < HC (p-value)** |
| --- | --- | --- | --- | --- |
| Medial/orbital-frontal cortex | aMpf1_L | 0.836 | 0.249 | 0.426 |
|  | aMpf1_R | 0.492 | 0.123 | 0.659 |
|  | vlOrb1_L | 0.865 | 0.936 | 0.997 |
|  | vlOrb1_R | 0.962 | 0.998 | 0.993 |
|  | vlOrb2_L | 0.813 | 0.753 | 0.995 |
|  | vlOrb2_R | 0.904 | 0.904 | 0.977 |
|  | aMpf2_L | 0.917 | 0.766 | 0.970 |
|  | aMpf2_R | 0.992 | 0.739 | 0.972 |
|  | vMpf_L | 0.972 | 0.990 | 0.998 |
|  | vMpf_R | 0.991 | 0.987 | 0.974 |
|  | dlOrb_L | 0.665 | 0.524 | 0.966 |
|  | dlOrb_R | 0.536 | 0.480 | 0.492 |
| Insula | Hg_ins_L | 0.136 | 0.122 | 0.529 |
|  | Hg_ins_R | 0.015 | 0.115 | 0.017 |
|  | vAng_ins_L | 0.873 | 0.816 | 0.857 |
|  | vAng_ins_R | 0.679 | 0.463 | 0.536 |
|  | dAng_ins_L | 0.061 | 0.281 | 0.518 |
|  | dAng_ins_R | 0.325 | 0.202 | 0.007 |
|  | vD&G_ins_L | 0.235 | 0.318 | 0.549 |
|  | vD&G_ins_R | 0.041 | 0.012 | 0.146 |
|  | dGran_ins_L | 0.329 | 0.153 | 0.351 |
|  | dGran_ins_R | 0.167 | 0.245 | 0.006 |
|  | dDysg_ins_L | 0.172 | 0.459 | 0.550 |
|  | dDysg_ins_R | 0.116 | 0.615 | 0.041 |
| Cingulate | dCing_L | 0.852 | 0.313 | 0.860 |
|  | dCing_R | 0.826 | 0.423 | 0.474 |
|  | rvCing_L | 0.939 | 0.970 | 0.979 |
|  | rvCing_R | 0.509 | 0.750 | 0.861 |
|  | Pregen_L | 0.216 | 0.249 | 0.174 |
|  | Pregen_R | 0.710 | 0.023 | 0.055 |
|  | vCing_L | 0.815 | 0.830 | 0.927 |
|  | vCing_R | 0.723 | 0.834 | 0.936 |
|  | cd_Cing_L | 0.182 | 0.234 | 0.238 |
|  | cd_Cing_R | 0.666 | 0.377 | 0.493 |
|  | cCing_L | 0.002 | 0.002 | 0.313 |
|  | cCing_R | 0.107 | 0.198 | 0.345 |
|  | Subgen_L | 0.596 | 0.135 | 0.340 |
|  | Subgen_R | 0.565 | 0.109 | 0.174 |
| Amygdala | mAmyg_L | 0.920 | 0.716 | 0.892 |
|  | mAmyg_R | 0.902 | 0.669 | 0.456 |
|  | lAmyg_L | 0.533 | 0.541 | 0.649 |
|  | lAmyg_R | 0.183 | 0.535 | 0.274 |
| Hippocampus | aHipp_L | 0.490 | 0.813 | 0.866 |
|  | aHipp_R | 0.824 | 0.915 | 0.844 |
|  | pHipp_L | 0.698 | 0.939 | 0.964 |
|  | pHipp_R | 0.997 | 0.998 | 0.964 |
| Basal Ganglia | vCaud_L | 0.599 | 0.306 | 0.397 |
|  | vCaud_R | 0.446 | 0.250 | 0.105 |
|  | GP_L | 0.105 | 0.172 | 0.050 |
|  | GP_R | 0.475 | 0.313 | 0.035 |
|  | NAC_L | 0.277 | 0.361 | 0.098 |
|  | NAC_R | 0.975 | 0.999 | 0.964 |
|  | vmPut_L | 0.130 | 0.055 | 0.063 |
|  | vmPut_R | 0.235 | 0.206 | 0.040 |
|  | dCaud_L | 0.405 | 0.250 | 0.314 |
|  | dCaud_R | 0.604 | 0.811 | 0.833 |
|  | dlPut_L | 0.052 | 0.299 | 0.045 |
|  | dlPut_R | 0.080 | 0.059 | 0.029 |
| Thalamus | mPFtha_L | 0.439 | 0.412 | 0.016 |
|  | mPFtha_R | 0.803 | 0.220 | 0.018 |
|  | mPMtha_L | 0.823 | 0.906 | 0.408 |
|  | mPMtha_R | 0.033 | 0.204 | 0.025 |
|  | Stha_L | 0.305 | 0.598 | 0.059 |
|  | Stha_R | 0.176 | 0.762 | 0.084 |
|  | rTtha_L | 0.165 | 0.753 | 0.303 |
|  | rTtha_R | 0.115 | 0.709 | 0.138 |
|  | Pptha_L | 0.038 | 0.742 | 0.148 |
|  | Pptha_R | 0.265 | 0.233 | 0.431 |
|  | Octha_L | 0.813 | 0.555 | 0.604 |
|  | Octha_R | 0.678 | 0.649 | 0.693 |
|  | cTtha_L | 0.255 | 0.442 | 0.396 |
|  | cTtha_R | 0.436 | 0.448 | 0.911 |
|  | lPFtha_L | 0.236 | 0.197 | 0.128 |
|  | lPFtha_R | 0.075 | 0.276 | 0.056 |

**Table S7**. Cohen’s D effect sizes for regional participation results per contrast. Highlighted values represent significant results (red = FDR corrected, yellow = uncorrected).

|  | | | **Effect size (Cohen's D)** | | | |
| --- | --- | --- | --- | --- | --- | --- |
| **Graph measure** | **Structure** | **sub-region** | **SUDEP > HC** | **High-risk > HC** | **SUDEP > low-risk** | **SUDEP > high-risk** |
| Participation | Medial/orbital-frontal cortex | aMpf1_L | 0.498 | 0.443 | 0.513 | 0.123 |
|  |  | aMpf1_R | 0.298 | 0.588 | 0.151 | 0.199 |
|  |  | vlOrb1_L | 0.724 | 0.522 | 0.548 | 0.137 |
|  |  | vlOrb1_R | 0.267 | 0.539 | 0.014 | 0.228 |
|  |  | vlOrb2_L | 1.054 | 0.913 | 0.303 | 0.186 |
|  |  | vlOrb2_R | 0.475 | 0.978 | 0.045 | 0.528 |
|  |  | aMpf2_L | 0.652 | 0.757 | 0.127 | 0.013 |
|  |  | aMpf2_R | 0.140 | 0.555 | 0.041 | 0.318 |
|  |  | vMpf_L | 0.579 | 0.479 | 0.038 | 0.172 |
|  |  | vMpf_R | 0.874 | 0.495 | 0.471 | 0.282 |
|  |  | dlOrb_L | 0.971 | 0.434 | 0.594 | 0.514 |
|  |  | dlOrb_R | 0.189 | 0.862 | 0.034 | 0.614 |
|  | Insula | Hg_ins_L | 1.053 | 0.887 | 0.283 | 0.057 |
|  |  | Hg_ins_R | 0.110 | 0.320 | 0.347 | 0.243 |
|  |  | vAng_ins_L | 1.181 | 1.343 | 0.233 | 0.187 |
|  |  | vAng_ins_R | 0.349 | 0.513 | 0.134 | 0.148 |
|  |  | dAng_ins_L | 0.447 | 0.883 | 0.119 | 0.444 |
|  |  | dAng_ins_R | 0.668 | 0.677 | 0.265 | 0.159 |
|  |  | vD&G_ins_L | 0.555 | 0.921 | 0.230 | 0.407 |
|  |  | vD&G_ins_R | 0.857 | 0.756 | 0.073 | 0.163 |
|  |  | dGran_ins_L | 0.619 | 1.139 | 0.235 | 0.533 |
|  |  | dGran_ins_R | 0.848 | 0.889 | 0.448 | 0.029 |
|  |  | dDysg_ins_L | 0.305 | 1.125 | 0.432 | 0.654 |
|  |  | dDysg_ins_R | 0.568 | 0.798 | 0.163 | 0.275 |
|  | Cingulate | dCing_L | 0.623 | 0.512 | 0.558 | 0.255 |
|  |  | dCing_R | 0.598 | 0.490 | 0.437 | 0.065 |
|  |  | rvCing_L | 1.031 | 0.665 | 0.248 | 0.321 |
|  |  | rvCing_R | 0.694 | 0.771 | 0.064 | 0.104 |
|  |  | Pregen_L | 0.782 | 0.770 | 0.129 | 0.100 |
|  |  | Pregen_R | 0.881 | 0.170 | 0.508 | 0.552 |
|  |  | vCing_L | 0.300 | 0.256 | 0.310 | 0.038 |
|  |  | vCing_R | 0.608 | 0.310 | 0.264 | 0.110 |
|  |  | cd_Cing_L | 0.726 | 0.430 | 0.158 | 0.245 |
|  |  | cd_Cing_R | 0.752 | 0.653 | 0.277 | 0.170 |
|  |  | cCing_L | 0.149 | 0.330 | 0.302 | 0.217 |
|  |  | cCing_R | 1.050 | 0.315 | 0.018 | 0.713 |
|  |  | Subgen_L | 0.888 | 0.602 | 0.693 | 0.159 |
|  |  | Subgen_R | 0.766 | 0.486 | 0.129 | 0.161 |
|  | Amygdala | mAmyg_L | 1.278 | 0.795 | 0.684 | 0.486 |
|  |  | mAmyg_R | 1.070 | 0.562 | 0.296 | 0.373 |
|  |  | lAmyg_L | 1.024 | 0.442 | 0.432 | 0.519 |
|  |  | lAmyg_R | 0.104 | 0.141 | 0.291 | 0.263 |
|  | Hippocampus | aHipp_L | 0.782 | 0.966 | 0.285 | 0.062 |
|  |  | aHipp_R | 1.045 | 0.851 | 0.621 | 0.355 |
|  |  | pHipp_L | 0.811 | 0.405 | 0.551 | 0.561 |
|  |  | pHipp_R | 1.251 | 0.883 | 0.850 | 0.600 |
|  | Basal Ganglia | vCaud_L | 0.260 | 0.544 | 0.019 | 0.299 |
|  |  | vCaud_R | 0.456 | 0.117 | 0.788 | 0.325 |
|  |  | GP_L | 0.273 | 0.341 | 0.246 | 0.026 |
|  |  | GP_R | 0.895 | 0.649 | 0.635 | 0.347 |
|  |  | NAC_L | 0.588 | 0.576 | 0.410 | 0.011 |
|  |  | NAC_R | 0.679 | 0.711 | 0.409 | 0.003 |
|  |  | vmPut_L | 0.972 | 0.685 | 0.380 | 0.227 |
|  |  | vmPut_R | 1.168 | 0.457 | 0.600 | 0.353 |
|  |  | dCaud_L | 0.159 | 0.340 | 0.153 | 0.147 |
|  |  | dCaud_R | 0.496 | 1.084 | 0.446 | 0.522 |
|  |  | dlPut_L | 0.116 | 0.611 | 0.203 | 0.559 |
|  |  | dlPut_R | 0.187 | 0.459 | 0.346 | 0.274 |
|  | Thalamus | mPFtha_L | 1.351 | 0.340 | 0.696 | 0.727 |
|  |  | mPFtha_R | 1.617 | 0.299 | 1.199 | 1.550 |
|  |  | mPMtha_L | 0.876 | 0.786 | 0.605 | 0.190 |
|  |  | mPMtha_R | 0.637 | 0.507 | 0.039 | 0.072 |
|  |  | Stha_L | 1.157 | 0.556 | 0.076 | 0.524 |
|  |  | Stha_R | 0.869 | 0.140 | 0.066 | 0.630 |
|  |  | rTtha_L | 0.121 | 0.693 | 0.455 | 0.638 |
|  |  | rTtha_R | 1.031 | 0.661 | 0.470 | 0.856 |
|  |  | Pptha_L | 0.986 | 0.961 | 0.218 | 0.001 |
|  |  | Pptha_R | 1.605 | 0.370 | 0.650 | 0.890 |
|  |  | Octha_L | 1.171 | 0.861 | 0.493 | 0.510 |
|  |  | Octha_R | 0.986 | 0.662 | 0.602 | 0.439 |
|  |  | cTtha_L | 0.993 | 0.514 | 0.170 | 0.480 |
|  |  | cTtha_R | 0.987 | 0.499 | 0.264 | 0.283 |
|  |  | lPFtha_L | 0.529 | 0.494 | 0.107 | 0.039 |
|  |  | lPFtha_R | 0.849 | 0.379 | 0.388 | 0.396 |

**Table S8**. Cohen’s D effect sizes for regional increases and decreases in degree centrality per contrast. Values highlighted in red indicate increased significant results, while values in green represent decreases (based on FDR-corrected two- sample t-tests results as per tables S5 and S6 above).

|  | | | **Effect size (Cohen's D)** | | |
| --- | --- | --- | --- | --- | --- |
| **Graph measure** | **Structure** | **sub-region** | **SUDEP v HC** | **High-risk v HC** | **Low-risk v HC** |
| **Degree centrality** | Medial/orbital-frontal cortex | aMpf1_L | 0.533 | 0.106 | 0.133 |
|  |  | aMpf1_R | 0.031 | 0.352 | 0.279 |
|  |  | vlOrb1_L | 0.549 | 0.693 | 1.222 |
|  |  | vlOrb1_R | 0.846 | 1.080 | 1.018 |
|  |  | vlOrb2_L | 0.470 | 0.367 | 1.035 |
|  |  | vlOrb2_R | 0.530 | 0.446 | 0.740 |
|  |  | aMpf2_L | 0.728 | 0.334 | 0.809 |
|  |  | aMpf2_R | 1.186 | 0.246 | 0.674 |
|  |  | vMpf_L | 0.888 | 0.789 | 1.163 |
|  |  | vMpf_R | 1.041 | 0.687 | 0.635 |
|  |  | dlOrb_L | 0.187 | 0.067 | 0.867 |
|  |  | dlOrb_R | 0.223 | 0.278 | 0.334 |
|  | Insula | Hg_ins_L | 0.587 | 0.528 | 0.090 |
|  |  | Hg_ins_R | 0.959 | 0.411 | 0.793 |
|  |  | vAng_ins_L | 0.426 | 0.207 | 0.207 |
|  |  | vAng_ins_R | 0.206 | 0.047 | 0.037 |
|  |  | dAng_ins_L | 0.712 | 0.294 | 0.158 |
|  |  | dAng_ins_R | 0.056 | 0.183 | 0.943 |
|  |  | vD&G_ins_L | 0.317 | 0.233 | 0.005 |
|  |  | vD&G_ins_R | 0.863 | 0.906 | 0.530 |
|  |  | dGran_ins_L | 0.352 | 0.482 | 0.230 |
|  |  | dGran_ins_R | 0.513 | 0.265 | 1.042 |
|  |  | dDysg_ins_L | 0.559 | 0.192 | 0.206 |
|  |  | dDysg_ins_R | 0.479 | 0.112 | 0.703 |
|  | Cingulate | dCing_L | 0.458 | 0.106 | 0.520 |
|  |  | dCing_R | 0.391 | 0.002 | 0.153 |
|  |  | rvCing_L | 0.563 | 0.571 | 0.723 |
|  |  | rvCing_R | 0.028 | 0.297 | 0.587 |
|  |  | Pregen_L | 0.340 | 0.237 | 0.226 |
|  |  | Pregen_R | 0.156 | 0.786 | 0.504 |
|  |  | vCing_L | 0.333 | 0.410 | 0.771 |
|  |  | vCing_R | 0.262 | 0.410 | 0.805 |
|  |  | cd_Cing_L | 0.446 | 0.299 | 0.152 |
|  |  | cd_Cing_R | 0.174 | 0.086 | 0.147 |
|  |  | cCing_L | 1.353 | 1.149 | 0.213 |
|  |  | cCing_R | 0.598 | 0.809 | 0.027 |
|  |  | Subgen_L | 0.167 | 0.303 | 0.027 |
|  |  | Subgen_R | 0.204 | 0.290 | 0.078 |
|  | Amygdala | mAmyg_L | 0.420 | 0.200 | 0.453 |
|  |  | mAmyg_R | 0.430 | 0.107 | 0.101 |
|  |  | lAmyg_L | 0.057 | 0.013 | 0.047 |
|  |  | lAmyg_R | 0.361 | 0.056 | 0.244 |
|  | Hippocampus | aHipp_L | 0.160 | 0.243 | 0.293 |
|  |  | aHipp_R | 0.370 | 0.495 | 0.365 |
|  |  | pHipp_L | 0.216 | 0.618 | 0.672 |
|  |  | pHipp_R | 1.175 | 1.160 | 0.670 |
|  | Basal Ganglia | vCaud_L | 0.074 | 0.246 | 0.159 |
|  |  | vCaud_R | 0.062 | 0.078 | 0.301 |
|  |  | GP_L | 0.605 | 0.230 | 0.519 |
|  |  | GP_R | 0.062 | 0.098 | 0.675 |
|  |  | NAC_L | 0.277 | 0.116 | 0.531 |
|  |  | NAC_R | 0.937 | 1.291 | 0.700 |
|  |  | vmPut_L | 0.477 | 0.563 | 0.648 |
|  |  | vmPut_R | 0.249 | 0.225 | 0.669 |
|  |  | dCaud_L | 0.137 | 0.307 | 0.188 |
|  |  | dCaud_R | 0.157 | 0.302 | 0.319 |
|  |  | dlPut_L | 0.812 | 0.225 | 0.770 |
|  |  | dlPut_R | 0.411 | 0.465 | 0.666 |
|  | Thalamus | mPFtha_L | 0.051 | 0.128 | 0.943 |
|  |  | mPFtha_R | 0.373 | 0.416 | 1.019 |
|  |  | mPMtha_L | 0.344 | 0.475 | 0.108 |
|  |  | mPMtha_R | 0.761 | 0.269 | 0.806 |
|  |  | Stha_L | 0.137 | 0.122 | 0.566 |
|  |  | Stha_R | 0.254 | 0.277 | 0.524 |
|  |  | rTtha_L | 0.585 | 0.065 | 0.425 |
|  |  | rTtha_R | 0.636 | 0.002 | 0.565 |
|  |  | Pptha_L | 0.717 | 0.280 | 0.329 |
|  |  | Pptha_R | 0.312 | 0.310 | 0.190 |
|  |  | Octha_L | 0.379 | 0.076 | 0.068 |
|  |  | Octha_R | 0.212 | 0.082 | 0.171 |
|  |  | cTtha_L | 0.480 | 0.248 | 0.382 |
|  |  | cTtha_R | 0.186 | 0.170 | 0.233 |
|  |  | lPFtha_L | 0.853 | 0.281 | 0.449 |
|  |  | lPFtha_R | 0.462 | 0.589 | 0.588 |

**Table S9.** Hub prevalence across groups. Means and standard deviations of the number of hubs per group are shown, along with group comparison effect sizes (Cohen’s *D*).

| **Group** | Mean number of hubs | Standard deviation | Effect size V SUDEP | Effect size V high-risk | Effect size V low-risk | Effect size V HC |
| --- | --- | --- | --- | --- | --- | --- |
| HC | 13.25 | 2.7 | 0.46 | 0.16 | 0.4 |  |
| Low-risk | 14.13 | 1.5 | 1.22 | 0.7 |  |  |
| High-risk | 12.88 | 1.9 | 0.39 |  |  |  |
| SUDEP | 12.13 | 1.8 |  |  |  |  |

**Table S10.** Regulatory regions of interest selected from the Harvard-Oxford cortical and subcortical atlas to build secondary analysis subnetwork. L = left, R = right.

| **ROI number** | **Anatomical label** |
| --- | --- |
| 1 | Insular_Cortex_L |
| 2 | Insular_Cortex_R |
| 3 | Frontal_Medial_Cortex_L |
| 4 | Frontal_Medial_Cortex_R |
| 5 | Subcallosal_Cortex_L |
| 6 | Subcallosal_Cortex_R |
| 7 | Cingulate_Gyrus_anterior_division_L |
| 8 | Cingulate_Gyrus_anterior_division_R |
| 9 | Cingulate_Gyrus_posterior_division_L |
| 10 | Cingulate_Gyrus_posterior_division_R |
| 11 | Frontal_Orbital_Cortex_L |
| 12 | Frontal_Orbital_Cortex_R |
| 13 | Parahippocampal_Gyrus_anterior_division_L |
| 14 | Parahippocampal_Gyrus_anterior_division_R |
| 15 | Parahippocampal_Gyrus_posterior_division_L |
| 16 | Parahippocampal_Gyrus_posterior_division_R |
| 17 | Brain-Stem_L |
| 18 | Brain-Stem_R |
| 19 | Thalamus_L |
| 20 | Thalamus_R |
| 21 | Caudate_L |
| 22 | Caudate_R |
| 23 | Putamen_L |
| 24 | Putamen_R |
| 25 | Pallidum_L |
| 26 | Pallidum_R |
| 27 | Hippocampus_L |
| 28 | Hippocampus_R |
| 29 | Amygdala_L |
| 30 | Amygdala_R |
| 31 | Accumbens_L |
| 32 | Accumbens_R |

1. **Supplementary Figures**


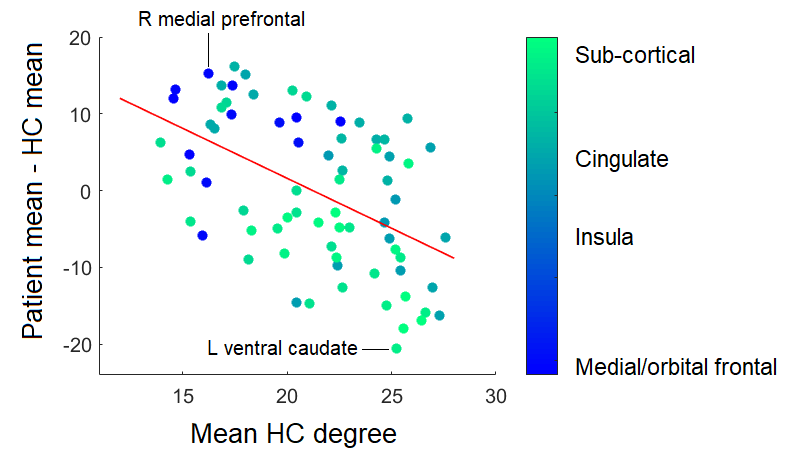


**Figure S1.** Hub distribution index calculated for one subject. Scatter showing individual patient mean minus the HC mean on the y axis, and mean degree of healthy controls on the x axis. Red line is the least squares regression line fitted to the data, showing a slope of -0.78 in this example.

**
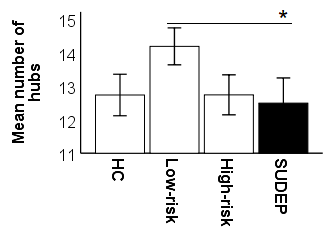
**

**Figure S2.** Bar graph demonstrating greater number of hubs among the subnetwork in low-risk patients, accounting for mean connectivity. HC = healthy control, * = *p* < 0.05. Error bars = standard error mean +/- 1.


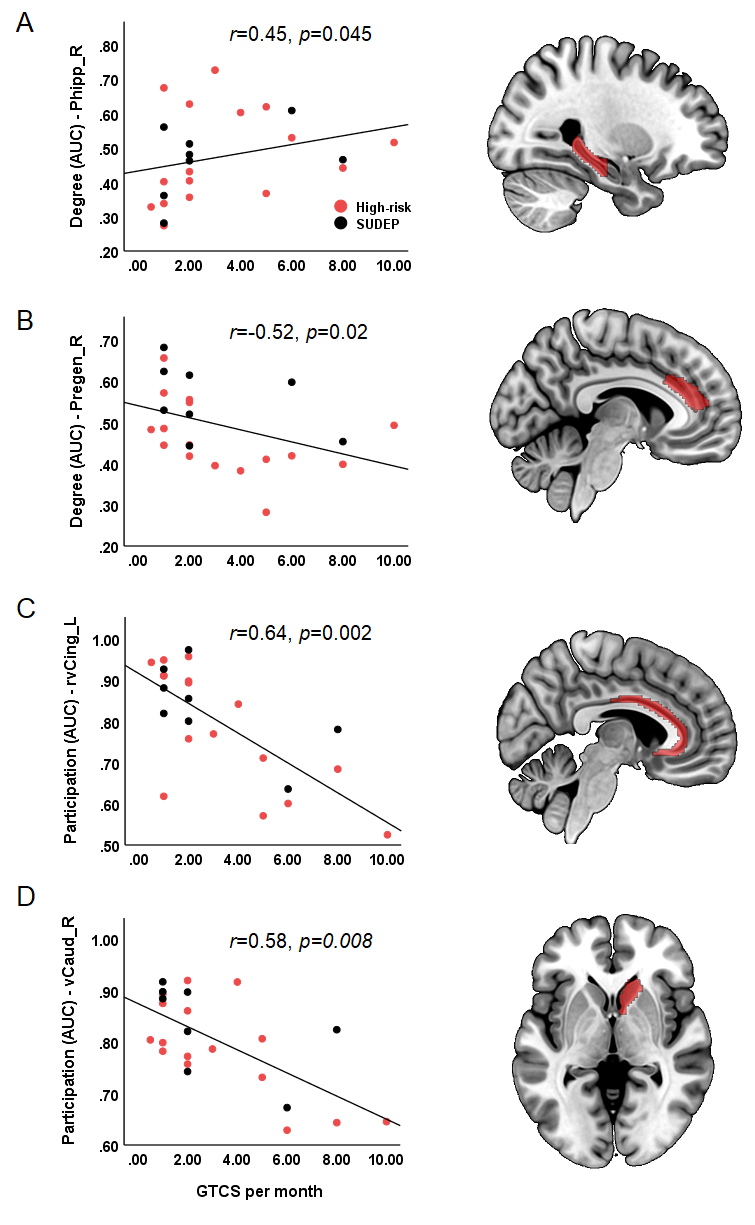


**Figure S3.** Significant correlations between GTCS frequency and network measures degree (A and B) and participation (C and D). Right: Related sites; right posterior hippocampus (A), right pregenual cingulate (B), rostroventral cingulate (C), and right ventral caudate (D). P-values were FDR corrected.


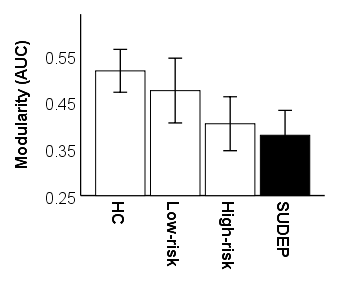


**Figure S4.** Bars showing reduced (*p* > 0.05) subnetwork modularity in patients when analysis was performed using the Harvard-Oxford atlas. Although not significant, reduced modularity followed the same pattern as that revealed using the Brainnetome atlas.

**Supplementary references**

Achard, S., Delon-Martin, C., Vértes, P. E., Renard, F., Schenck, M., Schneider, F., ... & Bullmore, E. T. (2012). Hubs of brain functional networks are radically reorganized in comatose patients. Proceedings of the National Academy of Sciences, 109(50), 20608-20613.

Achard, S., Salvador, R., Whitcher, B., Suckling, J., & Bullmore, E. D. (2006). A resilient, low-frequency, small-world human brain functional network with highly connected association cortical hubs. Journal of Neuroscience, 26(1), 63-72.

Alexander-Bloch, A. F., Gogtay, N., Meunier, D., Birn, R., Clasen, L., Lalonde, F., ... & Bullmore, E. T. (2010). Disrupted modularity and local connectivity of brain functional networks in childhood-onset schizophrenia. Frontiers in systems neuroscience, 4, 147.

Ashburner, J. (2007). A fast diffeomorphic image registration algorithm. Neuroimage, 38(1), 95-113.

Blondel, V. D., Guillaume, J. L., Lambiotte, R., & Lefebvre, E. (2008). Fast unfolding of communities in large networks. Journal of statistical mechanics: theory and experiment, 2008(10), P10008.

Bullmore, E., Fadili, J., Maxim, V., Şendur, L., Whitcher, B., Suckling, J., ... & Breakspear, M. (2004). Wavelets and functional magnetic resonance imaging of the human brain. Neuroimage, 23, S234-S249.

Fan, L., Li, H., Zhuo, J., Zhang, Y., Wang, J., Chen, L., ... & Fox, P. T. (2016). The human brainnetome atlas: a new brain atlas based on connectional architecture. Cerebral cortex, 26(8), 3508-3526.

Kruskal, J. B. (1956). On the shortest spanning subtree of a graph and the traveling salesman problem. Proceedings of the American Mathematical society, 7(1), 48-50.

Percival, D. B., & Walden, A. T. (2000). Wavelet methods for time series analysis, vol. 4 of Cambridge Series in Statistical and Probabilistic Mathematics.

Power, J. D., Barnes, K. A., Snyder, A. Z., Schlaggar, B. L., & Petersen, S. E. (2012). Spurious but systematic correlations in functional connectivity MRI networks arise from subject motion. Neuroimage, 59(3), 2142-2154.

Prim, R. C. (1957). Shortest connection networks and some generalizations. Bell system technical journal, 36(6), 1389-1401.

Rubinov, M., & Sporns, O. (2010). Complex network measures of brain connectivity: uses and interpretations. Neuroimage, 52(3), 1059-1069.

Song, X. W., Dong, Z. Y., Long, X. Y., Li, S. F., Zuo, X. N., Zhu, C. Z., ... & Zang, Y. F. (2011). REST: a toolkit for resting-state functional magnetic resonance imaging data processing. PloS one, 6(9), e25031.

Yan, C., & Zang, Y. (2010). DPARSF: a MATLAB toolbox for" pipeline" data analysis of resting-state fMRI. Frontiers in systems neuroscience, 4, 13.

Guimera R, Amaral LA. Functional cartography of complex metabolic networks. nature. 2005;433(7028):895.
